# Clinical features and outcomes of neuroleptospirosis in French Guiana: Insights from a retrospective study (2015–2021)

**DOI:** 10.1371/journal.pntd.0014263

**Published:** 2026-10-15

**Authors:** Audrey Delahaye, Tanguy Dequidt, Fabrice Quet, Clémentine Montagnac, Theo Blaise, Hatem Kallel, Felix Djossou, Mathieu Nacher, Kinda Schepers, Julien Coussement, Paul Le Turnier, Loïc Epelboin

**Affiliations:** 1 Department of Infectious and Tropical Diseases, University Hospital of French Guiana, Cayenne, French Guiana; 2 Clinical Investigation Centre of French Guiana, Inserm CIC 1424, University Hospital of French Guiana, Cayenne, French Guiana; 3 Inserm UA17, Amazonian Institute of Population Health, Cayenne, French Guiana; 4 Department of Infectious and Tropical Diseases, University Hospital of Guadeloupe, Les Abymes, Guadeloupe; 5 Department of Neurology, University Hospital of French Guiana, Cayenne, French Guiana; 6 Intensive Care Unit, University Hospital of French Guiana, Cayenne, French Guiana; University of Kentucky College of Medicine, UNITED STATES OF AMERICA

## Abstract

**Background:**

Leptospirosis is a re-emerging public health threat in French Guiana with a broad clinical spectrum. Neurological involvement (neuroleptospirosis) lacks a consensus definition and may be underrecognized. This study aimed to characterize its epidemiology, clinical features, outcomes, and associated factors.

**Methods:**

A multicenter retrospective study was conducted across healthcare facilities in French Guiana from 2015 to 2021. Consecutive hospital-managed patients with laboratory-confirmed or probable leptospirosis were included. Neuroleptospirosis was defined by neurological symptoms combined with objective cerebrospinal fluid (CSF) and/or neuro-imaging abnormalities. Patients with and without neurological involvement were compared.

**Results:**

Among 146 patients, 18 (12%) met the criteria for neuroleptospirosis, corresponding to a mean annual incidence of 0.88 cases/100,000 inhabitants (95% conference interval [CI], 0.52–1.39). Presentations were meningeal (78% [14/18]), encephalitic (17% [3/18]) or meningoencephalitic (6% [1/18]). Lumbar puncture was performed in 28/146 (19%) patients; pleocytosis was observed in 18/28 (64%). The median CSF leukocyte count was 42 cells/mm^3^ (interquartile range [IQR], 13–240 cells/mm^3^), with lymphocytic predominance in 8/13 differential counts (62%). CSF protein was mildly elevated (median, 0.51 g/L; IQR, 0.32–0.88), without hypoglycorrhachia. Brain magnetic resonance imaging (MRI) revealed abnormalities in 2/8 patients with neuroleptospirosis: one cerebral infarction and one case of pachymeningitis. Age under 30 years (odds ratio [OR], 5.56; 95% CI, 1.72–18), absence of leukocytosis during hospitalization (OR, 3.29; 95% CI, 1.04–10.39), and absence of thrombocytopenia (OR, 4.46; 95% CI, 1.39–14.32) were associated with neuroleptospirosis. Under our diagnostic criteria, neuroleptospirosis cases were associated with favorable outcomes, with lower odds of sepsis (OR, 0.28; 95% CI, 0.10–0.79), no intensive care unit admissions or deaths.

**Conclusions:**

This study clarifies the clinical spectrum and outcomes of neuroleptospirosis in French Guiana, highlighting the importance of clinical awareness in tropical endemic settings. Prospective studies incorporating systematic CSF analysis and microbiological testing are needed to improve understanding of its pathophysiology and standardize diagnostic pathways.

## Author summary

Leptospirosis is a re-emerging public health threat in French Guiana, with diverse clinical presentations. Its neurological manifestations (neuroleptospirosis), for which no consensus definition exists, remain underrecognized. Accordingly, a multicenter retrospective study was conducted, enrolling consecutive hospital-managed patients with laboratory-supported leptospirosis to characterize the epidemiological features of neuroleptospirosis and identify factors associated with neurological involvement. We applied a standardized composite definition based on neurological symptoms combined with cerebrospinal fluid analysis and/or neuro-imaging. Neuroleptospirosis accounted for approximately one tenth (12%) of hospitalized leptospirosis cases, predominantly presenting as meningitis (78%). CSF profiles typically showed moderate lymphocytic pleocytosis, closely mimicking viral meningitis. Strikingly, according to our strict diagnostic criteria, neuroleptospirosis cases exhibited fewer severity outcomes (less frequent sepsis, no intensive care admissions, and zero mortality) compared with non-neurological forms. In an exploratory multivariate analysis, younger age (< 30 years) and the absence of both leukocytosis and thrombocytopenia during hospitalization were significantly associated with a diagnosis of neuroleptospirosis. Neuroleptospirosis accounted for a non-negligible proportion of leptospirosis cases in French Guiana, with CSF features mimicking viral meningitis and favorable outcomes under antibiotic therapy. Increasing awareness of neuroleptospirosis and improving its recognition are essential to reduce underdiagnosis and refine patient management in endemic tropical settings.

## Introduction

Leptospirosis is a globally distributed bacterial zoonosis caused by pathogenic spirochaetes of the genus *Leptospira* [1]. In French Guiana, the disease is endemic and its incidence has significantly increased in recent years, with the mean annual rate rising from 5.8 to 14.0 per 100,000 adults between 2007–2014 and 2016–2022 [2,3]. The clinical presentation of leptospirosis is highly heterogeneous and nonspecific, ranging from mild, self-limited illness to life-threatening disease [1]. While classical clinical forms are well described, atypical manifestations may lead to delayed diagnosis or misdiagnosis [1].

Neurological involvement, referred to as neuroleptospirosis, remains poorly recognized and is likely underdiagnosed among central nervous system (CNS) infections [4,5]. In patients presenting primarily with neurological symptoms associated with fever, bacterial or viral meningitis are often considered the most likely diagnosis [4]. Leptospires are susceptible *in vitro* to most beta-lactams commonly used empirically in suspected bacterial meningitis [4,6]. In addition, *Leptospira* polymerase chain reaction (PCR) testing is not included in cerebrospinal fluid (CSF) multiplex panels [1,7]. As a result, the diagnosis of leptospirosis can be missed, even when patients receive antibiotics that effectively treat the infection and are administered empirically for suspected bacterial meningitis [8–10]. Therefore, neuroleptospirosis may be overlooked [1,4–7,11,12]. Differential diagnosis is particularly challenging in tropical regions, where multiple infections can affect the CNS, including arboviruses and malaria [13]. In South America, several studies conducted in Brazil have retrospectively reported leptospirosis as a common cause of aseptic meningitis (defined by clinical signs and symptoms of meningitis with pleocytosis but a negative Gram stain and sterile bacterial cultures) [6,12].

A wide spectrum of neurological complications has been associated with leptospirosis, including meningitis, encephalitis, stroke, Guillain-Barré syndrome, acute disseminated encephalomyelitis, myelitis or cranial nerve palsies, and uveitis [4–6,8,10,14–21]. Neuroleptospirosis is especially prone to underdiagnosis or misdiagnosis when neurological manifestations are isolated, without concomitant systemic features suggestive of leptospirosis [1,7,11,18]. Delayed recognition may delay the initiation of appropriate treatment and adversely affect outcomes [1,22]. Improved characterization of the clinical and laboratory features of neuroleptospirosis is therefore essential to enhance physician awareness and diagnostic accuracy [6].

To address the lack of clinical data and consensus definitions, the primary objective of this study was to characterize the epidemiology, clinical features and outcomes of neuroleptospirosis. The secondary objective was to identify the factors associated with neurological involvement in leptospirosis. By applying a standardized definition, our study aimed to characterize the clinical spectrum of neuroleptospirosis in French Guiana and identifies specific factors associated with this presentation.

## Methods

### Ethics statement

The study was registered with the Health Data Hub (No. F20220426211656). All patients were informed about their right to object to the use of their data through a personalized letter accompanied by an objection form, supplemented by informational posters displayed across all participating clinical departments. This retrospective study relied exclusively on routinely collected healthcare data and involved no intervention or additional biological sample collection. The study complied with the French reference methodology MR-004 for research using health data without direct involvement of participants and was declared to the French Data Protection Authority (Commission Nationale de l’ Informatique et des Libertés, CNIL) under the above registration number. The data used in this study were de-identified prior to analysis, and all procedures complied with applicable ethical standards.

### Study design

This multicenter study was conducted across all the public healthcare facilities in the territory, i.e., the three public hospitals of French Guiana (Cayenne, Kourou, and Saint-Laurent-du-Maroni), including the 17 hospital-affiliated remote prevention and care centers (Centres Délocalisés de Prévention et de Soins, CDPS), as well as the three community hospitals serving most of the inland municipalities, between January 2015 and December 2021. All consecutive cases of leptospirosis managed in hospital settings that met the inclusion criteria during the study period were included. Patients managed exclusively in outpatient primary care settings (i.e., general practitioners) were not included. Exclusion criteria were age under 18 years at the time of diagnosis, patient refusal to participate, or incomplete medical records.

### Inclusion criteria

In accordance with World Health Organization (WHO) and Centers for Disease Control and Prevention (CDC) guidelines, the diagnosis of leptospirosis was based on the presence of compatible clinical features, in the absence of a more likely alternative diagnosis, combined with microbiological evidence [23,24]. Microbiological evidence of leptospirosis was defined as either a positive PCR result (in serum, urine, or CSF), and/or a positive anti-*Leptospira* immunoglobulin M (IgM) serology (performed via enzyme-linked immunosorbent assay [ELISA] technique, with or without confirmation by microscopic agglutination test (MAT) [3,25]. MAT was performed only after an initially positive IgM serology result.

Leptospirosis cases were classified as confirmed or probable according to predefined diagnostic criteria (S1 Table).

**Confirmed** leptospirosis was defined by either a positive PCR result (serum, urine, or CSF) or by serological criteria combining an IgM level ≥ 50 IU/mL with a MAT titer ≥ 1:200, an IgM seroconversion with a MAT titer ≥ 1:200, a MAT seroconversion to a titer of ≥ 1:400, or at least a fourfold increase in MAT titer. Alternatively, a confirmed case could be defined by two IgM levels ≥ 100 IU/mL taken at least seven days apart.

**Probable** leptospirosis was defined, in patients presenting with compatible clinical symptoms and no alternative diagnosis, by an IgM level ≥ 50 IU/mL or IgM seroconversion with either MAT ≥ 1:100 (and < 1:200), MAT seroconversion ≥ 1:100 (and < 1:400), or a less than fourfold increase in MAT titer. Alternatively, a probable case could be defined by a single IgM level ≥ 100 IU/mL if MAT was unavailable.

**Neuroleptospirosis** was defined by (i) a confirmed or probable diagnosis of leptospirosis associated with (ii) neurological signs and (iii) abnormal CSF findings and/or brain or spinal imaging abnormalities attributable to leptospirosis. The first day of the illness was defined as the first day on which symptoms appeared (regardless of the presence of fever or neurological signs). Neurological syndromes were classified according to the guidelines of the French Infectious Diseases Society (SPILF) [26]. A meningeal syndrome was defined by the presence of at least two of the following symptoms: headache, neck stiffness, photophonophobia, or vomiting. An encephalitic syndrome was defined by seizures, impaired consciousness, or focal neurological deficits; patients fulfilling both definitions were classified as having a meningoencephalitic syndrome. CSF abnormalities were defined using standard thresholds: pleocytosis as a white cell count ≥ 5 cells/mm^3^ (the CSF leukocyte differential was performed at the discretion of the laboratory physician), elevated protein concentration as ≥ 0.45 g/L and hypoglycorrhachia as CSF glucose < 40% of the concomitant blood glucose level. Additional CSF parameters were interpreted according to local laboratory reference ranges, including lactate (1.1–2.4 mmol/L) and IgG (10–30 mg/L). Brain and spinal imaging abnormalities were defined based on the interpreting radiologist’s report.

Severe leptospirosis was defined as the need for vasopressors or mechanical ventilation, initiation of renal replacement therapy, or outcome of death [2]. Severity outcomes were assessed throughout the entire hospital stay. The Sequential Organ Failure Assessment (SOFA) score was retrospectively calculated when data were available, with sepsis defined as a SOFA score ≥ 2 [27]. Acute Respiratory Distress Syndrome (ARDS) was defined according to Berlin criteria [28]. Antibiotic therapy considered active against leptospirosis included β-lactams (excluding aztreonam), tetracyclines, macrolides, fluoroquinolones, and aminoglycosides [29].

### Microbiological analyses

Biological samples were analyzed as part of routine clinical care in the region at Cerba Laboratory and Biomnis Laboratory (mainland France). Both laboratories performed in-house real-time PCR assays targeting the *Leptospira* 16S rRNA (ribosomal ribonucleic acid) and *lipL32* genes, as well as anti-*Leptospira* IgM serology using an ELISA test (Serion, Germany). ELISA results were interpreted as positive for titers ≥ 50 IU/mL, intermediate for titers between 15 and 50 IU/mL, and negative for titers ≤ 15 IU/mL [30,31]. MAT was performed either at Eurofins Biomnis or at the French National Reference Center for Leptospirosis (Institut Pasteur, Paris), using a standard serogroup panel. The full panel of reference antigens used for the MAT is provided in S2 Table. The presumptive infecting serogroup was defined by the highest MAT titer detected in the most recent serum sample; identical titers across multiple serogroups were classified as co-agglutinins.

### Data collection and study procedures

Patients were identified using International Classification of Diseases, 10^th^ Revision (ICD-10) codes from participating healthcare centers, cross-referenced with laboratory results. Subsequently, electronic medical records were manually screened for eligibility. For included patients, demographic, exposure, clinical, laboratory, imaging, management and outcome data were retrospectively collected using a standardized, pseudonymized case report form. Baseline clinical and non-clinical findings were obtained within the first 48 hours of the initial healthcare encounter and the maximum and minimum laboratory values recorded during hospitalization were also documented.

### Statistical analysis

Quantitative variables were summarized as medians and interquartile ranges, and qualitative variables as frequencies and percentages with *n* indicating the number of available observations for each variable. No imputation was performed for missing data. Comparisons of quantitative variables were made using Student’s t-test for normally distributed variables or the Mann-Whitney *U* test for non-normally distributed variables or when the sample size was < 40. To preserve statistical power, bivariate comparisons of age and laboratory parameters were performed using their raw continuous values. Continuous variables were categorized exclusively for exploratory multivariable logistic regression analyses to provide clinically interpretable odds ratios. Continuous variables, including laboratory parameters, were categorized using clinically relevant thresholds reported in the literature or, when available, according to the observed median or mean, as appropriate to the distribution.

Qualitative variables were compared using the chi-squared test or Fisher’s exact test. All tests were two-tailed, and p-values ≤ 0.05 were considered statistically significant. Statistical analyses were performed using Stata version 19 (StataCorp LLC, College Station, TX, USA).

An exploratory multivariable analysis was performed using logistic regression to identify factors associated with neuroleptospirosis. Due to the limited number of events (*n* = 18 cases of neuroleptospirosis), the multivariable model was strictly restricted to key variables of interest to minimize overfitting and must be interpreted solely as exploratory and hypothesis-generating. A backward stepwise approach was applied, initially including all variables with p ≤ 0.20 in bivariate analyses, while excluding redundant variables. Variable selection was guided by the Akaike information criterion (AIC), with variables retained if their removal increased the AIC. Model fit was assessed using the Hosmer-Lemeshow goodness-of-fit test. Incidence rates of hospital-managed neuroleptospirosis per 100,000 inhabitants per year were estimated using population data from the National Institute of Statistics and Economic Studies (INSEE).

## Results

Over the 7-year study period, 146 consecutive hospital-managed patients with leptospirosis were identified, of whom 18 patients (12%) met the study criteria for neuroleptospirosis (Fig 1). The annual incidence of hospital-managed neuroleptospirosis was 0.88 per 100,000 inhabitants per year (95% CI 0.52–1.39). The sociodemographic characteristics, comorbidities and epidemiological exposures of patients with and without neuroleptospirosis are presented in Table 1. Patients with neuroleptospirosis had a median age of 26 years (interquartile range IQR 23–35). The male-to-female ratio was 5:1. Half of patients were born in Brazil (8/16, 50%), and 25% in French Guiana (4/16). Most patients had at least one at-risk exposure, including occupational (80%), environmental (67%), or animal-related (80%) exposures.

**Table 1:** Sociodemographic characteristics, comorbidities and epidemiological exposures among patients with leptospirosis in French Guiana (2015–2021).

Characteristics	Neuroleptospirosis (*n* = 18)	Non-neuroleptospirosis (*n* = 128)	p-value
**Age, years**	26 [23–35]	38 [31–54]	0.001*
18–29	11 (61)	29 (23)	
30–59	7 (39)	77 (60)	
≥ 60	0 (0)	22 (17)	
**Male sex**	15 (83)	99 (77)	0.764
**Place of birth**			
Haiti	2/16 (13)	37/104 (36)	0.087
Brazil	8/16 (50)	24/104 (23)	**0.033**
French Guiana	4/16 (25)	18/104 (17)	0.491
Other places of birth^1^	2/16 (13)	25/104 (24)	–
**Place of residence**			**0.008**
Cayenne & surrounding areas	9/17 (53)	103/124 (83)	–
Other municipalities	8/17 (47)	21/124 (17)	–
**Any comorbidity**	7 (39)	40 (31)	0.995
Hypertension	3 (17)	17 (13)	0.715
Diabetes	2 (11)	12 (9)	0.684
Obesity	3 (17)	10 (8)	0.304
HIV infection	2 (11)	2(2)	0.074
**Substance use**	7 (39)	49 (38)	0.961
Current smoking	3 (17)	30 (23)	0.523
Chronic alcohol use	3 (17)	19 (15)	0.841
Illicit drug use	2 (11)	17 (13)	0.626
**Any neurological condition**	2/18 (11)	17/123 (14)	0.360
Stroke	2 (11)	3 (2)	0.115
Migraine	0 (0)	2 (2)	–
Epilepsy	0 (0)	1 (1)	–
Other neurological condition^2^	0 (0)	11 (9)	–
**Occupational exposure**	8/14 (57)	62/102 (61)	0.780
Construction work	4 (29)	22 (22)	
Informal employment	0 (0)	10 (10)	
Gold mining	3 (21)	6 (6)	
Other at-risk occupations^3^	1 (7)	24 (24)	
Non-at-risk occupations^4^	3 (21)	25 (25)	
Unemployed	3 (21)	15 (15)	
**Environmental exposure**	10/15 (67)	86/104 (83)	0.164
Proximity to forests	8 (53)	46 (44)	
Swimming in freshwater	5 (33)	33 (32)	
Precarious living conditions	2 (13)	21 (20)	
Other at-risk exposures^5^	2 (13)	38 (37)	
**Animal exposure**	12/15 (80)	77/91 (85)	0.705
Rodents	8 (53)	55 (60)	
Dogs	4 (27)	17 (19)	
Other animals	4 (27)	23 (25)	

**Table 1** presents a comparative analysis of baseline socio-demographic factors and environmental, animal, and occupational exposures between patients diagnosed with neuroleptospirosis (NL, *n* = 18) and those with presumed non-neuroleptospirosis (NNL, *n* = 128).

**Data Presentation:** Quantitative variables are expressed as medians and interquartile ranges (IQR). Qualitative variables are expressed as frequencies and percentages (n/N, %).

**Statistical Analysis:** P-values were calculated using Student’s t-test or Mann-Whitney *U* test for continuous variables (including age as a continuous variable*), and the chi-squared test or Fisher’s exact test for categorical variables. Bold P-values indicate statistical significance (p ≤ 0.05).

**Footnote Definitions:**

^**1**^Other places of birth: mainland France (*n* = 9), Guyana (*n* = 7), Dominican Republic (*n* = 4), Suriname (*n* = 3), French West Indies (*n* = 3), China (*n* = 1).

^**2**^Other neurological conditions: subdural hematoma (*n* = 2), pneumococcal meningoencephalitis (*n* = 1), idiopathic tremor (*n* = 1), sciatica (*n* = 1), complex regional pain syndrome (*n* = 1), vertigo (*n* = 1), forearm nerve injury complicated by claw-like finger deformity and fingertip paresthesia (*n* = 1), facial paralysis (*n* = 1), Guillain-Barré syndrome (*n* = 1), cerebral toxoplasmosis (*n* = 1).

^**3**^Other at-risk occupations: maintenance worker (*n* = 8), fisherman (*n* = 7), military personnel (*n* = 3), plumber (*n* = 3), farmer (*n* = 1), logger (*n* = 1), boatman (*n* = 1), slaughterhouse worker (*n* = 1).

^**4**^Non-at-risk occupations: occupations other than building work, “little jobs”, gold mining, other at-risk occupations or unemployment.

^**5**^Other at-risk exposures: flooding, gardening, recreational fishing, consumption of non-potable water, and water sports (canoeing and kayaking).

**Abbreviations**: **BMI**, body mass index; **HIV**, human immunodeficiency virus; **IQR**, interquartile range; **NL**, neuroleptospirosis; **NNL**, non-neuroleptospirosis.

**Fig 1:**
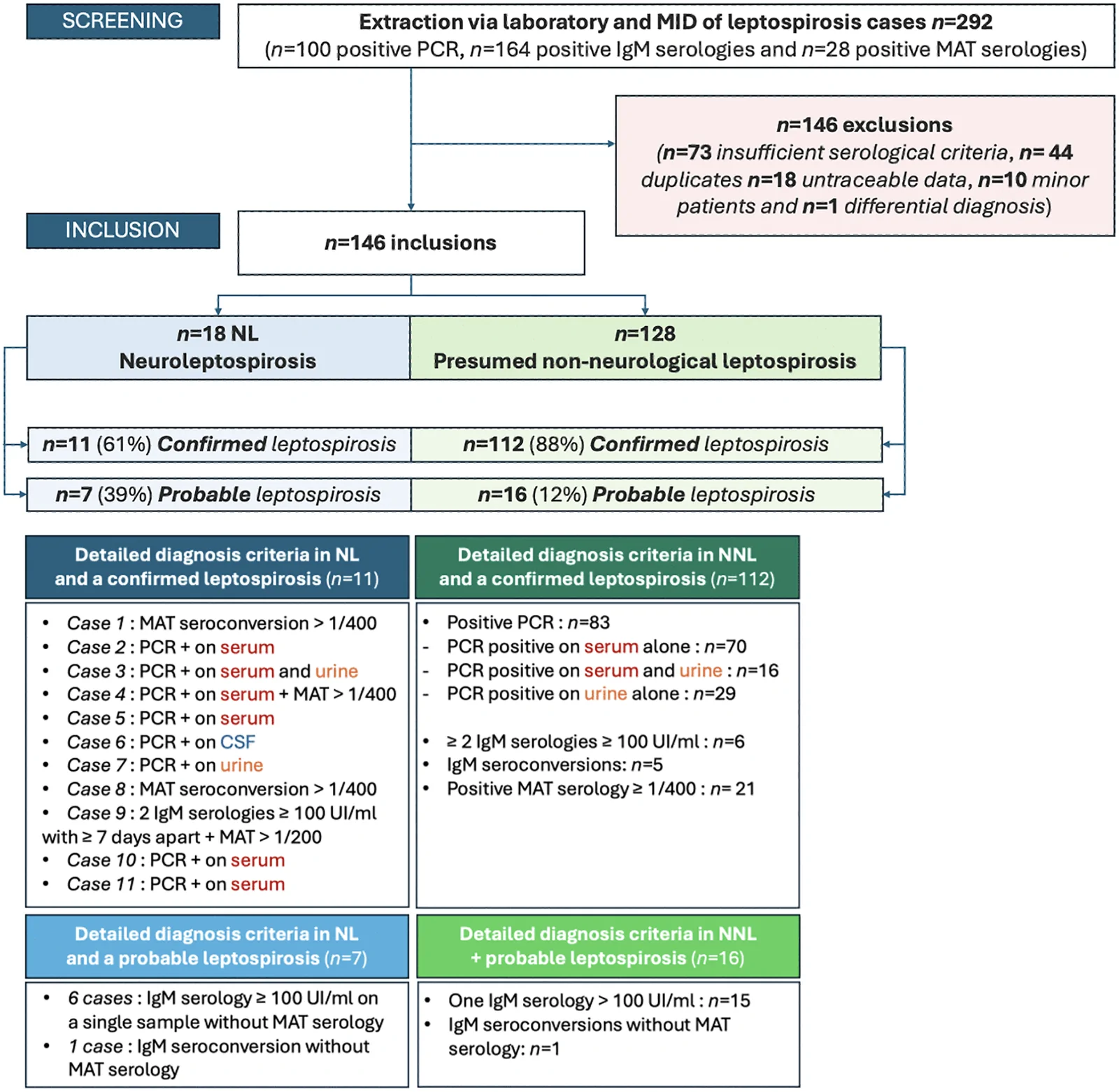
Flowchart of patient selection and diagnostic classification. The diagram illustrates the study selection process, starting with the initial extraction of 292 suspected cases from laboratory and Medical Information Department (MID) records, resulting in the final inclusion of 146 patients. Following the exclusion of 146 records, patients were categorized into two distinct groups: **neuroleptospirosis (NL, *n =* 18)** and **presumed non-neurological leptospirosis (NNL, *n* = 128)**. **Definitions of Diagnostic categories:** • **Neuroleptospirosis case (NL):** presence of (i) confirmed or probable leptospirosis (as defined above), together with (ii) neurological signs, and abnormal CSF findings and/or brain or spinal imaging abnormalities attributable to leptospirosis. • **Confirmed leptospirosis:** a positive PCR result (serum, urine, and/or CSF); an IgM level ≥ 50 IU/mL; or IgM seroconversion*, with each of the latter two criteria also requiring either a MAT titer ≥ 1:200, a MAT seroconversion** ≥ 1:400, or a fourfold increase in MAT titer. Alternatively, a confirmed case could be defined by two IgM levels ≥ 100 IU/mL obtained at least seven days apart. • **Probable leptospirosis:** an IgM level ≥ 50 IU/mL or IgM seroconversion*, together with either MAT ≥ 1:100 (and < 1:200), MAT seroconversion ≥ 1:100 (and < 1:400), or a less than fourfold increase in MAT titer. Alternatively, a probable case could be defined by a single IgM level ≥ 100 IU/mL if MAT was unavailable. **Kinetic definitions:** • * **IgM seroconversion:** negative or equivocal IgM level (15–50 IU/mL) on the first sample, followed by a positive result (level ≥ 50 IU/mL) on the second sample collected at least seven days later. • ** **MAT seroconversion:** initial MAT < 1:100 on the first sample, followed by a positive result in a second sample collected at least seven days later. **Abbreviations: CSF**, cerebrospinal fluid; **MID**, Medical Information Department; **IgM**, immunoglobulin M; **MAT**, microscopic agglutination test; **NL**, neuroleptospirosis case; **NNL**, presumed non-neuroleptospirosis case; **PCR**, polymerase chain reaction.

Clinical presentations, antibiotic treatment, laboratory parameters, and severity outcomes in patients with and without neuroleptospirosis are presented in Table 2. In patients with neuroleptospirosis, neurological symptoms were generally present on the first day of illness (*n* = 16, 89%), and always during the first week, prior to laboratory confirmation. Regarding involvement of non-neurological organs, patients in the neuroleptospirosis group exhibited a mild systemic phenotype with low rates of visceral complications: 4/13 (31%) developed jaundice and only 1/18 (6%) presented with dyspnea. The clinical and laboratory characteristics (serum and CSF) of the 18 cases of neuroleptospirosis are presented in Part 1 of Table 3, while their diagnostic findings, neuro-imaging results, treatments, and clinical outcomes are presented in Part 2 of Table 3. Meningeal syndrome was the most frequent clinical presentation (*n* = 14, 78%). Isolated encephalitic syndrome occurred in three patients (17%), including two with impaired consciousness (minimum Glasgow coma score [GCS] of 9) and two with focal deficits: one with right hemiparesis and right central facial paralysis consistent with an ischemic stroke; the other initially presenting with febrile headaches, followed on day 5 by peripheral facial diplegia, dysarthria, dysphagia, and gait disturbances. Another patient (5.5%) was classified as having a meningoencephalitic syndrome, presenting with a meningeal syndrome associated with a sudden bilateral loss of visual acuity (blurred vision) on day 1. Peripheral nervous system symptoms were observed alongside CNS involvement in three patients (17%), consisting of distal paresthesia.

**Table 2:** Clinical presentations, antibiotic treatment, laboratory parameters, and severity outcomes of patients with and without neuroleptospirosis.

Characteristics	Neuroleptospirosis (*n* = 18)	Non-neuroleptospirosis (*n* = 128)	p-value
**Time between illness onset and the first healthcare encounter, days**	5.5 [3–8]	4 [2–6]	0.176
**Diagnostic classification**			**0.010**
Confirmed leptospirosis	11 (61)	112 (88)	
Probable leptospirosis	7 (39)	16 (12)	
**Duration of antibiotic therapy**	7 [7–10]	7 [7–18]	0.189
**Intravenous antibiotic therapy**	16/16 (100)	93/118 (79)	**0.042**
**Antibiotic therapy at meningeal doses**	10/17 (59)	0/115	**–**
**Length of hospital stay, days**	8.5 [6.5–13]	8 [5.5–11]	0.326
**Clinical data**			
Fever	10/17 (59)	60/124 (48)	0.450
Myalgia	12/12 (100)	86/90 (96)	0.877
Arthralgia	7/12 (58)	54/73 (74)	0.354
Cough	2/18 (11)	35/123 (29)	0.284
Dyspnea	1/18 (6)	25/123 (20)	0.332
Nausea and/or vomiting	13/17 (77)	61/119 (51)	0.130
Abdominal pain	12/18 (67)	61/126 (48)	0.392
Diarrhea	7/18 (39)	46/116 (40)	0.544
Jaundice	4/13 (31)	48/79 (61)	0.089
Bleeding	2/18 (11)	33/125 (26)	0.422
**Serum parameters**			
Hemoglobin, g/dL (*n* = 145)	12.8 [12.3–14.3]	12.8 [11.4–14]	0.414
WBC, x 10^9^/L (*n* = 143)	8.1 [5.7–11.3]	10.5 [8.1–13.9]	**0.030**
Platelet count, x 10^9^/L (*n* = 145)	200 [70–227]	118 [64–189]	0.147
ALT, U/L (*n* = 144)	30 [23–50]	46 [22–87]	0.249
AST, U/L (*n* = 134)	37 [30–55]	63 [30–107]	0.078
Total bilirubin, μmol/L (*n* = 139)	16 [10–41]	26 [12–104]	0.132
Creatinine, μmol/L (*n* = 135)	92 [71–120]	110 [87–207]	**0.048**
CRP, mg/L (*n* = 144)	169 [32–332]	210 [111–294]	0.700
Creatine kinase, U/L (*n* = 72)	312 [97–849]	525 [159–1227]	0.363
Lactate, mmol/L (*n* = 61)	1 [0.8–2.85]	1.9 [1.4–3]	0.127
**CSF parameters**			
WBC/mm^3^ (*n* = 20)	42 [13–240]	–	–
PMNs, % (*n* = 13)	25 [8–78]	–	–
Lymphocytes, % (*n* = 13)	68 [18–89]	–	–
Protein, g/L (*n* = 20)	0.51 [0.32–0.88]	–	–
Glucose, mmol/L (*n* = 20)	3.15 [3–3.66]	–	–
IgG, mg/L (*n* = 12)	86 [38–218]	–	–
Lactate, mmol/L (*n* = 14)	2.5 [1.7–3.2]	–	–
**Severity outcomes**			
ICU admission	0/18 (0)	21/127 (17)	–
SOFA score	1.5 [0–6]	4 [2–7]	**0.020**
Sepsis	9/18 (50)	95/122 (78)	**0.043**
Mechanical ventilation	0/18 (0)	9/127 (7)	–
ARDS	0/17 (0)	12/122 (10)	–
Vasopressors	0/18 (0)	13/126 (10)	–
Renal replacement therapy	0/18 (0)	9/127 (7)	–
Death	0/18 (0)	3/128 (2)	–

**Table 2** compares the clinical symptoms, serum biological parameters, and severity outcomes between the neuroleptospirosis (NL, *n* = 18) and non-neuroleptospirosis (NNL, *n* = 128). Clinical symptoms and serum biochemical parameters were assessed during the first 48 hours after hospital admission, whereas severity outcomes were assessed throughout the entire hospital stay.

**Data Presentation**: Quantitative variables are expressed as medians and interquartile ranges (IQR). Qualitative variables are expressed as frequencies and percentages (n/N, %). Bold p-values indicate statistical significance (p ≤ 0.05).

**Footnote Definitions**:

• **Fever:** Temperature ≥ 38°C at the first healthcare encounter, or two temperature measurements ≥ 38°C taken at least 30 minutes apart.

• **Jaundice:** Cutaneous and/or mucosal icterus, including isolated conjunctival icterus.

• **Illness onset:** The first day on which symptoms appeared (regardless of the presence of fever or neurological signs).

• **Severity outcomes:** assessed throughout the entire hospital stay.

• **Serum parameters:** Values recorded during the first 48 hours after the hospital encounter.

• **CSF parameters:** Data from all cerebrospinal fluid samples (*n* = 20) collected from patients with neuroleptospirosis (*n* = 18).

**Abbreviations**: **ALT**, alanine aminotransferase; **ARDS**, acute respiratory distress syndrome; **AST**, aspartate aminotransferase; **CRP**, C-reactive protein; **CSF**, cerebrospinal fluid; **ICU**, intensive care unit; **IgG**, immunoglobulin G; **PMNs**, polymorphonuclear neutrophils; **SOFA**, Sequential Organ Failure Assessment; **WBC**, white blood cell.

**Table 3:** Part 1: Clinical and laboratory characteristics (serum and CSF) in 18 patients with neuroleptospirosis.

Case	Age group	Sex	Detailed clinical manifestations suggestive of neuroleptospirosis	Sd	WBC count (x10^9^/L)	Platelet count (x10^9^/L)	Day of lumbar puncture^1^	CSF WBC count (cells/mm^3^)	N/L^2^	CSF Protein level (g/L)	CSF glucose level (mmol/L)	Serum glucose level (mmol/L)
1	26–30	Ma	Fever, headaches, and vomiting	M	6.4	219	10	1	-^3^	0.33	3.0	–
2	21–25	Ma	Fever, headaches, neck stiffness and pain, photophonophobia	M	2.1	70	2	14	90/10	0.41	3.8	–
3	26–30	Ma	Fever, headaches, vomiting, and paresthesia (tingling) in the right foot	M	12.2	18	2	14	-^5^	0.16	4.0	–
4	21–25	Ma	Fever, headaches, and vomiting	M	13.7	238	5	9	-^5^	0.27	4.0	8.2
5	21–25	Ma	Fever, headaches, and vomiting	M	8.9	30	8	35	25/67	0.93	3.1	6.0
6	31–35	Ma	Fever, headaches, neck stiffness, photophobia, and vomiting	M	4.27	57	7	12	76/21	0.31	3.1	6.1
7	46–50	Ma	Fever, acute right hemiparesis with brachio-facial predominance, right central facial paralysis, impaired consciousness with confusion (GCS 9–13)	E	10.6	68	4	5000	96/4	1.26	7.9	38.0
8	31–35	Ma	Fever, headaches, vomiting, neck stiffness, and dizziness	M	4.1	227	8	235	1/99	1.73	3.0	4.8
9	21–25	Ma	Fever, headaches, neck stiffness, and vomiting	M	7.2	227	15 17	800 240	20/80 10/90	0.82 0.33	2.9 3.1	5.1
10	31–35	Ma	Fever, headaches, and vomiting	M	13.7	183	4	48	-^5^	0.22	5.0	–
11	21–25	Fe	Fever, headaches, neck stiffness, and vomiting	M	10.2	348	2	1	-^4^	0.2	3.3	5.4
12	18–20	Ma	Fever, headaches, nausea, and photophonophobia	M	4.1	217	7	500	5/84	0.59	3.2	7.7
13	46–50	Ma	Fever, psychomotor slowing/drowsiness (GCS 14)	E	11.3	75	7	16	-^5^	1.1	3.5	–
14	21–25	Fe	Fever, headaches, photophonophobia, vomiting and painful paresthesia in the left leg	M	15.9	164	7	416	80/15	0.49	2.6	6.4
15	46–50	Ma	Fever, headaches, vomiting, transient sudden bilateral loss of visual acuity (blurred vision), paresthesia in all extremities, tinnitus, hypoesthesia in left foot	ME	10.2	222	10	240	13/87	0.94	3.2	–
16	21–25	Ma	Fever, headaches, neck stiffness, and vomiting	M	6.3	133	6	240	28/68	0.51	3.0	–
17	26–30	Ma	Fever, headaches, photophobia, and vomiting	M	6.9	304	6	200	51/40	0.59	2.4	5.2
18	46–50	Fe	Fever, headaches, peripheral diplegia, dysarthria, swallowing and gait disorders linked to motor deficit of the lower limbs	E	5.7	597	12 13	18 12	1/99 -^5^	0.75 0.50	2.9 3.3	4.9

Part 2: Diagnostic findings, neuro-imaging, treatment and clinical outcomes in 18 patients with neuroleptospirosis.				
Case	Diagnosis C/P^1^	Brain or spinal cord imaging (CT or MRI)	Antibiotic therapy	Clinical outcome
1	C - MAT seroconversion	–	Ceftriaxone 1g OD for 5D	Recovery without sequelae
2	C - PCR + on serum	Normal non-contrast brain CT	Ceftriaxone MD^2^ for 5D + Doxycycline 100mg BID for 7D	Recovery without sequelae
3	C - PCR + on serum	Normal non-contrast brain CT - Normal spinal cord MRI	Ceftriaxone 1g OD for 4D + Doxycycline 100mg BID for 7D	Survival, lost-to-follow-up
4	C - PCR + on serum	Normal contrast-enhanced brain CT	Ceftriaxone 1g OD + Doxycycline 100mg BID both for 10D	Survival, lost-to-follow-up
5	C - PCR + on serum	–	Cefotaxime MD for 7D	Survival, lost-to-follow-up
6	C - PCR + on CSF	Normal non-contrast brain CT	Ceftriaxone 2g OD for 7D + Rovamycin 3MIU TID for 5D	Recovery without sequelae
7	C - PCR + on urine	Normal non-contrast brain CT Brain MRI 1: Multiple supra and sub-tentorial ischemic lesions Brain MRI 2: Extensive ischemic lesion in the left superficial middle cerebral artery territory; thrombosis of the left middle cerebral artery	Ceftriaxone MD for 14D	Survival, lost-to-follow-up
8	C - MAT seroconversion	Normal contrast-enhanced brain CT	Ceftriaxone MD + Amoxicillin MD for 5D	Recovery without sequelae
9	C - 2 IgM tests > 100 UI/mL	–	Ceftriaxone MD for 10D + Amoxicillin MD for 14D	Recovery without sequelae
10	C - PCR + on serum	–	Piperacillin-tazobactam 4g TID + Vancomycin 1g BID + Metronidazole 500mg TID for 7D	Recovery without sequelae
11	C - PCR + on serum	–	Ceftriaxone 1g OD for 7D + Trimethoprim-sulfamethoxazole curative dose for 7D	Survival, lost-to-follow-up
12	P - 1 IgM test > 100 UI/L	–	None^3^	Recovery without sequelae
13	P - IgM test seroconversion^4^	Normal contrast-enhanced brain CT - Normal brain MRI	Ceftriaxone MD + Amoxicillin MD	Recovery without sequelae
14	P - 1 IgM test > 100 UI/L	–	Ceftriaxone MD + Amoxicillin MD for 4D then AMX 1g TID 3D	Recovery without sequelae
15	P - 1 IgM test > 100 UI/L	Normal non-contrast & contrast-enhanced brain CT Normal brain MRI	Amoxicillin MD for 6D then Amoxicillin 1g TID for 1D	Recovery without sequelae
16	P - 1 IgM test > 100 UI/L	Normal non-contrast brain CT	Ceftriaxone MD 2D + Amoxicillin MD 4D then AMX 1g TID 3D	Recovery without sequelae
17	P - 1 IgM test > 100 UI/L	Normal contrast-enhanced brain CT - Normal brain MRI	Ceftriaxone MD 2D + Amoxicillin MD 10D	Recovery without sequelae
18	P - 1 IgM test > 100 UI/L	Normal contrast-enhanced brain CT Brain MRI: Diffuse pachymeningitis - Normal spinal cord MRI	Ciprofloxacin 500mg BID for 3D	Survival with persistent facial diplegia

**Part 1 of Table 3** provides a detailed, case-by-case description of the clinical manifestations and laboratory findings (serum and cerebrospinal fluid [CSF]) for the 18 patients diagnosed with neuroleptospirosis included in the study.

**Footnote Definitions:**

**Clinical data:** Symptoms recorded at first healthcare encounter; **Serum data:** values recorded during the first 48 hours after hospital admission; **CSF data:** Results from all CSF samples (*n* = 20) collected from patients with neuroleptospirosis (*n* = 18).

^**1**^The day of lumbar puncture was calculated from the first day of illness, defined as the first day on which symptoms appeared, regardless of the presence of fever or neurological signs. ^**2**^When the sum of the percentages does not equal 100%, the remaining percentage corresponds to the proportion of monocytes. ^3^The elevated CSF lactate level (4 mmol/L; reference range: 1.1–2.4 mmol/L) was the biological criterion used, in combination with one or more clinical criteria, to classify the case as neuroleptospirosis. ^**4**^ The elevated CSF immunoglobulin G (IgG) level (32.7 mg/L; reference range: 10–30 mg/L) was the biological criterion used, in combination with one or more clinical criteria, to classify the case as neuroleptospirosis. No serum IgG measurement was available to calculate the CSF-to-serum IgG ratio. ^**5**^N/L was not performed because of an estimated insufficient pleocytosis at the discretion of the lab physician (despite the threshold of 5 cells/mm^3^ set by the French Infectious Diseases Society) in patients 3, 4 and 13, and because of a hematic macroscopic aspect in case 10.

**Part 2 of Table 3** presents the individual microbiological confirmation status, brain or spinal imaging findings, specific antibiotic regimens (including the regimen and duration), and clinical outcomes for the 18 cases of neuroleptospirosis.

**Footnote Definitions:**

^**1**^Diagnosis status: **C**, confirmed; **P**, probable, in accordance with the study’s standardized diagnostic algorithms (see S1 Table).

^**2**^Meningeal dose: high-dose intravenous antibiotic therapy tailored to achieve adequate central nervous system penetration (*e.g.*, ceftriaxone at 2 g once daily or penicillin at meningeal doses).

^**3**^Patient 12 did not receive antibiotic therapy because a viral meningitis was suspected; the patient recovered fully and spontaneously.

^**4**^Patient 13 could not be classified as having confirmed leptospirosis because MAT was not performed, so the diagnostic criteria could not be fulfilled.

**Abbreviations: BID**, twice daily (*bis in die*); **C**, confirmed diagnosis; **CSF**, cerebrospinal fluid; **CT**, computed tomography; **D**, day(s); **E**, encephalitic syndrome (defined by at least one of the following: seizures, impaired consciousness, focal neurological signs); **Fe**, Female; **GCS**, Glasgow coma scale; **IgM**, immunoglobulins M; **M**, meningeal syndrome (defined as at least two of the following: headaches, neck stiffness, vomiting or photophonophobia); **Ma**, male; M; **MAT**, microscopic agglutination test; **MD**, meningeal dose; **ME**, meningo-encephalitic syndrome (clinical presentation combining meningeal and encephalitic signs); **MIU**, million international units; **MRI**, magnetic resonance imaging; **N/L**, percentages of neutrophils/lymphocytes; **OD**, once daily (*omni die*); **P**, probable diagnosis; **Sd**, syndrome (meningeal, meningo-encephalitic or encephalitic); **TID**, three times daily (*ter in die*); **WBC**, white blood cells.

Laboratory parameters showed that patients with neuroleptospirosis did not exhibit leukocytosis (median 8.1 x 10^9^/L, IQR 5.7–11.3) or thrombocytopenia (median 200 x 10^9^/L, IQR 70–227). Only mild hepatic or renal abnormalities were present (Table 2). CSF analysis revealed pleocytosis in most patients with neuroleptospirosis (16/18, 89%), with a median cell count of 42 cells/mm³ (IQR 13–240). Detailed CSF parameters for samples from patients with neuroleptospirosis (*n* = 20/30, 67%) and those from patients without neuroleptospirosis (*n* = 10/30, 33%) are provided in S3 Table, with comparisons between the two groups presented in S4 Table. Among the 13 CSF differential counts available (only in the neuroleptospirosis group), the profile was predominantly lymphocytic (8/13, 62%). More specifically, during the first week of illness, 4/7 (57%) CSF samples showed a neutrophilic profile, 2/7 (29%) a lymphocytic profile and 1/7 (14%) a mixed profile. By contrast, from day 8 onward, all samples showed a lymphocytic profile, 6/6 (100%) (Tables 3 and S5). In addition, CSF analysis showed a mild elevation in protein concentration (median 0.51 g/L, IQR 0.32–0.88) and normal glucose levels. The limited number of MATs, following the withdrawal of reimbursement for this test in 2014, precluded an assessment of potential neurotropic strains (S6 Table).

Neuro-imaging data for patients with neuroleptospirosis (Tables 3) were available from brain imaging, including computed tomography (CT) and magnetic resonance imaging (MRI), as well as spinal cord MRI. Brain CT was performed in 11 patients (61%), brain MRI in 5 patients (28%), and spinal cord MRI in 2 (11%). All brain CT examinations (*n* = 11) and both spinal cord MRI examinations (*n* = 2) were normal. Of the five brain MRIs performed in addition to CT, two revealed acute abnormalities. One patient with peripheral facial diplegia showed diffuse pachymeningitis with bilateral facial nerve enhancement on MRI (Fig 2). Another patient presenting with stroke at admission had multiple recent ischemic lesions in the left middle cerebral artery territory, with a proximal thrombus.

**Fig 2:**
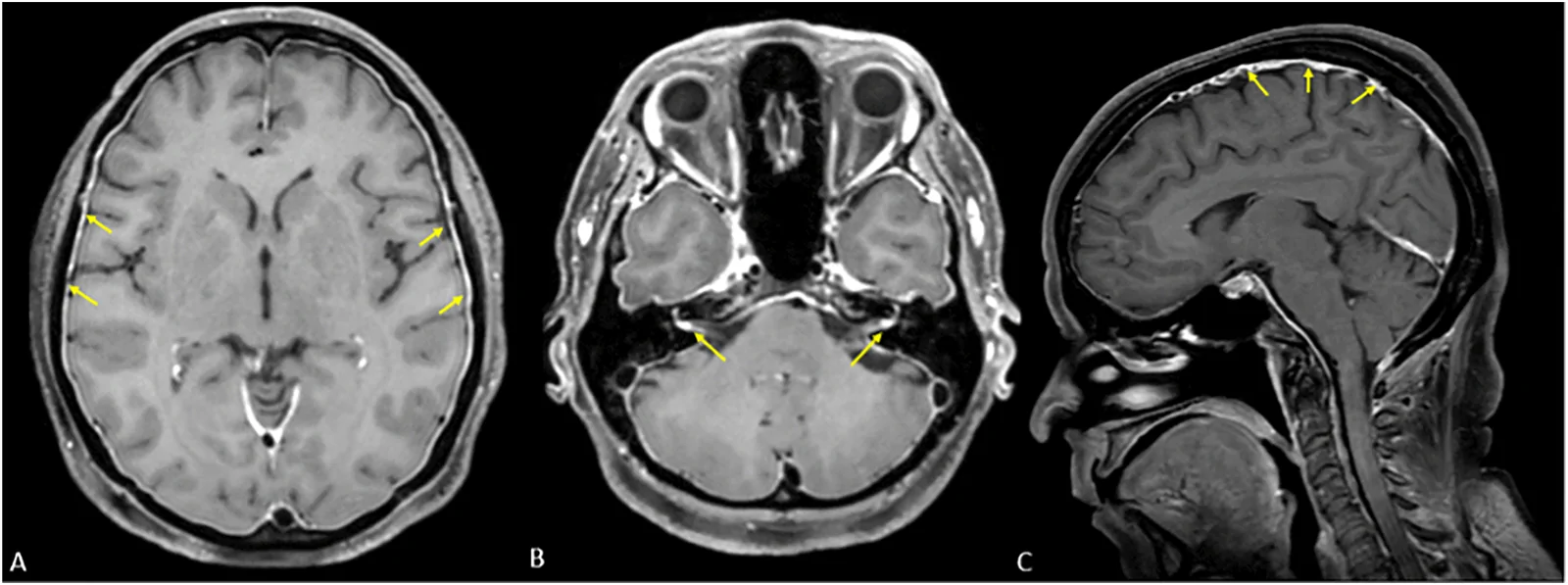
Magnetic resonance imaging (MRI) of case 18 demonstrating diffuse pachymeningitis and bilateral facial nerve enhancement (radiological interpretation by Dr Rasouly Najibullah). The multi-panel figure shows brain magnetic resonance imaging (MRI) findings of case 18, an adult patient who presented with acute meningeal symptoms followed by bilateral peripheral facial diplegia on day 5. The imaging findings are consistent with meningeal and cranial nerve inflammation in the context of neuroleptospirosis. **Panel Descriptions:** • **A. Axial T1-weighted turbo spin echo (TSE) sequence:** Demonstrates pachymeningeal thickening with prominent contrast enhancement. • **B. Axial T1-weighted TSE sequence:** Highlights bilateral, symmetrical enhancement of the facial nerves (cranial nerve VII) within the internal auditory canals, consistent with the patient’s clinical presentation of facial diplegia. • **C. Sagittal T1-weighted TSE sequence:** Shows extensive longitudinal pachymeningeal thickening and diffuse contrast enhancement. **Footnote Definitions:** • **Differential Diagnosis:** Alternative infectious and inflammatory causes of pachymeningitis, including neurosyphilis, Lyme neuroborreliosis, tuberculosis, and sarcoidosis, were investigated and reportedly excluded based on negative CSF PCR, culture, serologies, as well as serum angiotensin-converting enzyme levels. **Abbreviations: CSF**, cerebrospinal fluid; **MRI**, magnetic resonance imaging; **PCR**, polymerase chain reaction; **TSE**, turbo spin echo.

Regarding treatment, the median duration of antibiotic therapy was 7 days [IQR 7–10], with antibiotics frequently administered at meningeal doses (10/15, 67%). The most frequently used antibiotics were third-generation cephalosporins (14/17, 82%), penicillins (8/17, 47%), and doxycycline (3/17, 18%). Notably, no patients in either the neuroleptospirosis or the non-neuroleptospirosis group received adjunctive corticosteroid therapy during their hospital stay.

Regarding clinical outcomes in patients with neuroleptospirosis (Tables 2–3), none of the cases required admission to the intensive care unit (ICU) or died during their hospital stay. On day 5 after the onset of a febrile meningeal syndrome, one patient developed facial diplegia. This patient was lost to follow-up and had shown no improvement after one month of hospitalization. Among treated patients, 10/17 (59%) received antibiotics at meningeal doses and recovered without sequelae; 6/17 (35%) recovered despite receiving antibiotics at non-meningeal doses. One patient, in whom viral meningitis was suspected, recovered without antibiotic treatment.

Patients with and without a diagnosis of neuroleptospirosis were compared. Patients with neuroleptospirosis were younger (median age 26 *versus* [*vs.*] 38, p = 0.001), their white blood cell counts, and median creatinine levels were significantly lower (8.1 *vs.* 10.5 x 10^9^/L and 92 *vs.*110 μmol/L, respectively). Sepsis was less frequent, and SOFA scores were lower (p = 0.043 *vs*. p = 0.020) than in patients without neuroleptospirosis (Tables 1 and 2).

In an exploratory analysis, multivariate analysis suggested potential associations between a diagnosis of neuroleptospirosis and age under 30 years, absence of leukocytosis, and absence of thrombocytopenia (Table 4).

**Table 4:** Bivariate and exploratory multivariate logistic regression analyses of clinical and laboratory factors associated with a diagnosis of neuroleptospirosis.

	Bivariate analysis			Exploratory multivariate analysis		
Variable	Crude ORs	95% CI	p-value	Adjusted ORs	95% CI	p-value
Age < 30 years	5.36	[1.90–15.08]	0.001*	5.56	[1.72–18.00]	**0.004**
Confirmed diagnosis	0.22	[0.08–0.66]	0.007*	–	–	–
Dyspnea	0.23	[0.03–1.82]	0.164	–	–	–
Bleeding	0.35	[0.08–1.60]	0.175	–	–	–
Nausea-vomiting	3.09	[0.95–10.03]	0.060	–	–	–
SOFA Score	0.83	[0.70–0.99]	0.036*	–	–	–
Absence of leukocytosis	4.29	[1.49–12.32]	0.007*	3.29	[1.04–10.39]	**0.042**
Absence of thrombocytopenia	3.59	[1.29–9.94]	0.014*	4.46	[1.39–14.32]	**0.012**
AST < 60 U/L (2xULN)	2.41	[0.85–6.83]	0.097	–	–	–
ALT < 70 U/L (2xULN)	2.24	[0.70–7.2]	0.175	–	–	–
Absence of jaundice	2.48	[0.82–7.5]	0.107	–	–	–
Creatinine level < 130 μmol/L	4.15	[1.37–12.63]	0.012*	–	–	–

The table displays the crude and adjusted odds ratios (ORs) with their corresponding 95% confidence intervals (CI) to identify clinical and laboratory factors associated with neurological involvement in hospital-managed leptospirosis.

**Statistical Methodology:** Crude ORs correspond to the bivariate models. Adjusted ORs correspond to the exploratory multivariable model, which was developed using backward stepwise selection based on the Akaike information criterion (AIC). P-values in bold are statistically significant (p ≤ 0.05).

**Exploratory Nature Warning:** Due to the small number of events (*n* = 18 cases of neuroleptospirosis), this multivariable model is subject to overfitting and statistical instability, as reflected by the wide confidence intervals. The findings should be interpreted as exploratory and hypothesis-generating only.

**Footnote Definitions:**

• **Absence of leukocytosis:** Maximum white blood cell count recorded during hospitalization < 10 x 10^9^/L (local laboratory reference threshold).

• **Absence of thrombocytopenia:** Minimum platelet count recorded during hospitalization > 150 x 10^9^/L (local laboratory reference threshold).

• **Absence of jaundice:** Maximum total bilirubin level recorded during hospitalization < 40 μmol/L (local laboratory reference threshold).

• **Neuroleptospirosis case:** Presence of confirmed or probable leptospirosis associated with neurological signs and abnormal CSF and/or neuro-imaging findings.

**Abbreviations: ALT**, alanine aminotransferase; **AST**, aspartate aminotransferase; **CI**, confidence interval; **OR**, odds ratio; **SOFA**, Sequential Organ Failure Assessment; **ULN**, upper limit of normal.

## Discussion

This study provides a comprehensive description of the epidemiology, clinical presentation, and outcomes of neuroleptospirosis in French Guiana. Using stringent composite inclusion criteria based on clinical findings, CSF abnormalities and/or neuro-imaging findings, neurological involvement accounted for approximately one tenth of consecutive hospital-managed leptospirosis cases. This estimate is consistent with previous reports, which range from 5% to 20% of hospitalized cases [5,18,32]. However, our estimated annual incidence of 0.88 cases per 100,000 inhabitants reflects only hospital-managed neuroleptospirosis and must not be interpreted as the true population-level incidence. Milder cases with transient neurological symptoms (such as isolated headaches) managed entirely in outpatient settings would not have been captured by our study, meaning that the actual public health burden in French Guiana is likely higher. Nonetheless, previous studies indicate that most symptomatic leptospirosis cases in the territory are managed as inpatients (73–86%) [2,3]. Direct comparisons with other cohorts remain challenging because there is no consensus definition of neuroleptospirosis; some studies rely solely on clinical features, whereas others require CSF and/or radiological evidence [18,32].

The high proportions of patients born in Brazil and gold miners in the neuroleptospirosis group may reflect intense environmental and occupational exposure to *Leptospira* reservoirs in remote rainforest settings [33]. Differences in strain neurotropism, as reported for *Leptospira pomona*, or host genetic susceptibility cannot be excluded. However, we lacked molecular typing and patient genetic data to investigate these hypotheses [34]. Exposure associated with gold-mining activities therefore remains a plausible explanation for this finding.

In our series, neurological manifestations occurred early in the clinical course of illness, with 89% of cases experiencing neurological symptoms from day 1. This finding may be consistent with early neuroinvasion by leptospires during the initial bacteremic phase, as described in rodent models [35]. It challenges the classical biphasic model of human leptospirosis, which distinguishes the bacteremic phase from the subsequent immune phase. Under this model, neurological involvement may be interpreted as a delayed immune-mediated complication [36,37]. Meningeal presentations predominated, in line with findings from studies of patients managed on general hospital wards [18,32]. By contrast, intensive care cohorts have reported a higher prevalence of encephalitis [5,20]. This difference could reflect variation across the clinical spectrum related to delays in seeking care, referral biases, or distinct pathophysiological processes. Other neurological manifestations, including stroke and facial diplegia, were consistent with previous reports [5]. However, the patient presenting with stroke had multiple pre-existing cardiovascular risk factors (diabetes mellitus, arterial hypertension, and ischemic heart disease), suggesting that the stroke may have been coincidental rather than directly attributable to leptospirosis. For the patient with facial diplegia, the diagnosis of leptospirosis was classified as probable, as it relied solely on serological criteria. Several alternative diagnoses of facial diplegia were investigated and reportedly excluded, including Lyme disease, Guillain–Barré syndrome, human immunodeficiency virus [HIV], varicella-zoster virus infection, Epstein–Barr virus infection, and sarcoidosis.

Patients with neuroleptospirosis frequently lacked systemic leukocytosis and thrombocytopenia. Because these are common markers of the systemic inflammatory response, their absence suggests that early neurological presentations, particularly meningitic forms, may represent a less systemically inflammatory phenotype. Alternatively, the delay before hospital admission (median, 5.5 days from illness onset) may coincide with the natural evolution of the disease: normalization of leukocyte counts and platelet recovery. Although the clinical relevance of the significant differences in median white blood cells counts (8.1 *vs*. 10.5 x 10^9^/L) and creatinine (92 *vs*. 110 µmol/L) is debatable, the lower levels in the neuroleptospirosis group suggest a milder systemic phenotype. Notably, severe thrombocytopenia constitutes a major contraindication to lumbar puncture, which was mandatory for our case definition. This likely introduced selection bias toward milder hematological profiles by excluding the most severe systemic cases.

CSF findings were consistent with previous reports, typically showing moderate pleocytosis, lymphocytic predominance, mild protein elevation, and normal glucose levels [6,18,32,38]. A recent Brazilian review of 176 cases reported mean values of 68 cells/mm³ (range 30–7800), 73% lymphocytes, 18% neutrophils, normal glucose, and protein levels of 0.67–0.90 g/L. Our findings fall well within these ranges. Nevertheless, the wide dispersion observed in both cohorts demonstrates that neither marked pleocytosis nor early neutrophilic predominance should rule out the diagnosis of neuroleptospirosis [6]. Indeed, our temporal analysis showed a shift from neutrophilic or mixed profiles during the first week (57% of samples) to exclusively lymphocytic profiles from day 8 onward [38,39]. Both profiles remain compatible with neuroleptospirosis, and a normal or pauci-cellular CSF does not exclude it [4,40]. These findings suggest a dynamic local inflammatory response and emphasize that CSF profiles can closely mimic viral meningitis, underscoring the need to systematically consider leptospirosis in the differential diagnosis of aseptic meningitis in endemic tropical regions [11,41].

Regarding CSF PCR, it was not systematically performed in our study, due to local clinical practices. Previous studies have reported CSF PCR sensitivities ranging from 60% to 90% [42,43]. Leptospiral bacterial loads can be up to 10–fold higher in CSF than in blood, and CSF PCR may be positive despite a negative blood PCR [44]. The lack of systematic CSF PCR is an important microbiological limitation that may have led to missed early diagnoses, before pleocytosis developed or IgM levels rose. Including CSF PCR in future prospective protocols could improve early diagnostic sensitivity and help distinguish leptospirosis from viral meningitis. Serological diagnoses were significantly more frequent in neuroleptospirosis than in non-neurological cases, although PCR remained the primary diagnostic method. Rather than indicating a delayed immune phase (classical biphasic model), this finding may reflect delays in hospital presentation. With a median delay of 5.5 days between illness onset and admission to hospital, patients with neuroleptospirosis were often tested at the end of the first week of illness, during the transition period when blood PCR sensitivity may decline and anti-*Leptospira* IgM titers rise. The withdrawal of reimbursement for the MAT in France in 2014 may also have limited serological confirmation.

Regarding neuro-imaging, cerebral infarction has been previously reported in leptospirosis, but diffuse pachymeningitis is an exceptional finding mostly associated with other spirochetal infections like syphilis [20,45]. Alternative causes of pachymeningitis were investigated and reportedly excluded. These included syphilis, Lyme disease (serological testing), tuberculosis (CSF mycobacterial PCR and culture), granulomatosis with polyangiitis (anti-neutrophil cytoplasmic antibodies [ANCA]), and sarcoidosis (serum angiotensin-converting enzyme levels). Although post-lumbar puncture contrast leakage can cause dural enhancement, the bilateral facial nerve enhancement observed on MRI was consistent with the patient’clinical presentation of facial diplegia, supporting a possible association with neuroleptospirosis.

Neuroleptospirosis cases were significantly younger than non-neurological cases (median 26 *vs*. 38 years, p = 0.001), consistent with reports describing leptospiral meningitis as predominantly affecting young individuals [41,46]. Younger individuals may exhibit differences in endothelial function and blood–brain barrier permeability that facilitate early leptospiral neuroinvasion. Age-related effects on host susceptibility and disease presentation have also been described in other central nervous system infections and forms of meningitis [47,48].

The literature describes two contrasting prognostic profiles for neuroleptospirosis: favorable outcomes in isolated meningitic forms and high mortality in encephalitic forms, particularly those involving impaired consciousness [32,49]. In intensive care cohorts, neuroleptospirosis is often identified as an independent risk factor for death, with some severe cases directly attributable to severe neurological complications (cerebral edema, acute disseminated encephalomyelitis) [19,50,51]. In contrast, outcomes in our study were favorable, with no deaths or ICU admissions. This may reflect several factors, including young age, the predominance of meningeal presentations (78%) with limited involvement of other organs, and early treatment [22,51]. These meningeal forms generally have a more benign course than severe encephalitic presentations and fewer markers of systemic severity, including thrombocytopenia and hepatorenal dysfunction [52–54]. This milder presentation is consistent with findings from Sri Lanka and Laos, where neuroleptospirosis – particularly cases presenting as aseptic meningitis – was associated with a less severe clinical course and fewer markers of systemic severity [4,55]. In addition, all patients with neuroleptospirosis received intravenous antibiotics (*vs*. 79% in the non-neurological group, p = 0.042) and 59% received high, meningeal-dose regimens. This intensive treatment may have contributed to favorable outcomes. None of our patients received adjunctive corticosteroids, supporting the Cochrane systematic review’s conclusion that current evidence is insufficient to recommend routine steroid use [56]. However, this favorable prognosis must be interpreted cautiously. Our stringent case definition required CSF and/or neuro-imaging abnormalities. Critically ill patients with severe thrombocytopenia or shock may therefore have been excluded, potentially introducing selection and survivorship biases.

This study has several methodological limitations. First, its retrospective design introduces inherent information, selection and classification biases. By excluding outpatient cases and applying stringent objective inclusion criteria – which may not be met in very early presentations without CSF pleocytosis or when lumbar puncture is contraindicated (*e.g.,* severe thrombocytopenia) – our study may underestimate the true proportion of neuroleptospirosis. This artificially biased the prognosis towards a milder profile through selection and survivorship biases. Consequently, our findings reflect the clinical spectrum and prognosis of hospital-managed patients meeting our specific diagnostic criteria rather than the entire clinical spectrum of neuroleptospirosis.

Second, CSF PCR was not performed systematically. Although its sensitivity is imperfect, a positive result would have provided strong microbiological evidence of leptospiral neuroinvasion in probable cases and helped distinguish leptospirosis from alternate diagnoses, including viral meningitis.

Third, the classification of two patients as having neuroleptospirosis based on CSF abnormalities other than pleocytosis (elevated CSF lactate or IgG levels) may be debated. A sensitivity analysis excluding these cases was therefore performed; although it affected the multivariate model, crude odds ratios remained stable. In addition, the reliance on serology to define probable leptospirosis may be questioned; however, this limitation was mitigated using a high antibody-titer threshold (>100 IU/L).

Finally, the small sample size (*n* = 18 events) is a critical limitation. In multivariable logistic regression, standard guidelines recommend 10–15 events per variable to ensure model stability. With only 18 events and three retained predictors, our multivariate model is vulnerable to overfitting and statistical instability, as reflected by the wide 95% confidence intervals. Consequently, we have downgraded these multivariable findings to a strictly exploratory, hypothesis-generating analysis, and larger prospective cohorts are mandatory to validate these predictors with adequate statistical power.

## Conclusion

In this endemic tropical setting, neurological involvement accounted for approximately one tenth of hospital-managed leptospirosis cases, most often presenting as meningitis early in the course of illness. Neuroleptospirosis primarily affected younger patients and was associated with a less inflammatory biological profile, closely mimicking viral meningitis. Unlike reports from intensive care settings, neuroleptospirosis outcomes in our cohort were favorable, possibly reflecting the predominance of isolated meningitic forms. Distinguishing milder meningitic from severe encephalitic presentations may reconcile contrasting prognosis findings reported in the literature. Larger multicenter prospective cohorts with systematic CSF analysis and advanced microbiological testing are needed to further characterize the pathophysiology and standardize diagnostic pathways for neuroleptospirosis.

## Supporting information

S1 Table Diagnostic criteria for confirmed or probable leptospirosis. Detailed molecular (PCR) and serological (IgM ELISA and Microscopic Agglutination Test) thresholds and seroconversion kinetics used for classifying laboratory-confirmed or probable cases in the study cohort.
(DOCX)

S2 Table Reference antigen panel used for the microscopic agglutination test (MAT). List of the 25 antigens used as reference panels by the French National Reference Center for Leptospirosis (Institut Pasteur, Paris).
(DOCX)

S3 Table Cerebrospinal fluid parameters in 28 patients who underwent a lumbar puncture. Detailed individual cerebrospinal fluid (CSF) parameters of both neuroleptospirosis cases (*n =* 18) and non-neuroleptospirosis cases (*n* = 10).
(DOCX)

S4 Table Comparison of cerebrospinal fluid parameters between patients with and without neuroleptospirosis. Comparison of median values and interquartile ranges of CSF parameters between the neuroleptospirosis and non-neuroleptospirosis groups (*n* = 28).
(DOCX)

S5 Table Cerebrospinal fluid leukocyte predominance according to the timing of lumbar puncture in patients with neuroleptospirosis. Temporal evolution of neutrophilic, mixed, and lymphocytic predominance during the first and second weeks of illness (*n* = 18).
(DOCX)

S6 Table Distribution of MAT antibody titers and presumptive infecting serogroups. Distribution of MAT titers among the positive serologies in the study population.
(DOCX)
