# Supplementary material for: Clinical features and outcomes of neuroleptospirosis in French Guiana: Insights from a retrospective study (2015–2021)
Source: PLoS Negl Trop Dis. 2026 Oct 15;20(10):e0014263. doi: 10.1371/journal.pntd.0014263 (PMC13690270; doi:10.1371/journal.pntd.0014263)
Supplement: Supplementary file 1 [file pntd.0014263.s001.docx]

**S1 Table. Diagnostic criteria for confirmed or probable leptospirosis.**

| Diagnostic Status | Microbiological & Serological Criteria |
| --- | --- |
| Confirmed Leptospirosis | Presence of compatible clinical features in the absence of an alternative diagnosis, combined with: • Positive PCR (in serum, urine, and/or CSF) • Or IgM level ≥ 50 IU/mL or IgM seroconversion* AND at least one of the following:  - MAT titer ≥ 1:200  - MAT seroconversion** ≥ 1:400  - At least a fourfold increase in MAT titer; • Or two IgM ELISA tests ≥ 100 IU/mL taken at least seven days apart. |
| Probable Leptospirosis | Presence of compatible clinical features in the absence of a more likely alternative diagnosis, combined with: • IgM level ≥ 50 IU/mL or IgM seroconversion* AND at least one of the following:  - MAT titer ≥ 1:100 and < 1:200  - MAT seroconversion** ≥ 1:100 and < 1:400  - A less than fourfold increase in MAT titer; • Or a single IgM ≥ 100 IU/mL if MAT was unavailable. |

The table outlines the standardized diagnostic algorithm used to classify leptospirosis cases in the study cohort. It combines clinical compatibility with molecular (PCR) and serological (ELISA IgM and MAT) thresholds to define confirmed and probable cases.

**Kinetic definitions:**

* **IgM seroconversion:** negative or equivocal IgM result (15–50 IU/mL) on the first sample, followed by a positive result (≥ 50 IU/mL) on the second sample collected at least seven days later.

** **MAT seroconversion:** an initial MAT < 1:100 in the first sample, followed by a titer ≥ 1:100 in a second sample collected at least seven days later.

**Abbreviations:** **CSF**, cerebrospinal fluid; **ELISA**, enzyme-linked immunosorbent assay; **IgM**, immunoglobulin M; **MAT**, microscopic agglutination test; **PCR**, polymerase chain reaction
