# Supplementary material for: Clinical features and outcomes of neuroleptospirosis in French Guiana: Insights from a retrospective study (2015–2021)
Source: PLoS Negl Trop Dis. 2026 Oct 15;20(10):e0014263. doi: 10.1371/journal.pntd.0014263 (PMC13690270; doi:10.1371/journal.pntd.0014263)
Supplement: Supplementary file 2 [file pntd.0014263.s002.docx]

**S2 Table. Reference antigen panel used for the microscopic agglutination test (MAT).**

| Serogroups (25 antigens) | | | |
| --- | --- | --- | --- |
| *Australis* | *Autumnalis* | *Ballum* | *Bataviae* |
| *Canicola* | *Castellonis* | *Celledoni* | *Copenhageni* |
| *Cynopteri* | *Djasiman* | *Grippotyphosa* | *Hardjo* |
| *Hebdomadis* | *Icterohaemorrhagiae* | *Javanica* | *Louisiana* |
| *Mini* | *Panama* | *Pomona* | *Pyregenes* |
| *Sarmiento* | *Sejroe* | *Shermani* | *Tarassovi* |
| *Patoc* | | | |

The table displays the complete panel of 25 pathogenic antigens and one saprophytic control strain used by the French National Reference Center (NRC) for Leptospirosis (Institut Pasteur, Paris) to identify presumptive infecting serogroups.

**Note:** The *patoc* strain is a saprophytic, non-pathogenic strain used as a control.

**Abbreviations:** **MAT**, microscopic agglutination test.
