# Supplementary material for: Clinical features and outcomes of neuroleptospirosis in French Guiana: Insights from a retrospective study (2015–2021)
Source: PLoS Negl Trop Dis. 2026 Oct 15;20(10):e0014263. doi: 10.1371/journal.pntd.0014263 (PMC13690270; doi:10.1371/journal.pntd.0014263)
Supplement: Supplementary file 3 [file pntd.0014263.s003.docx]

**S3 Table. Cerebrospinal fluid parameters in 28 patients who underwent a lumbar puncture.**

| Day of lumbar puncture^1^ | CSF WBC count (cells/mm^3^) | CSF RBC count (cells/mm^3^) | N/L  (%) | CSF protein  level (g/L) | CSF glucose level (mmol/L) | Serum glucose level (mmol/L) |
| --- | --- | --- | --- | --- | --- | --- |
| Neuroleptospirosis cases (*n=18*) | | | | | | |
| D10 | 1 | 0 | - | 0.33 | 3.0 | - |
| D2 | 14 | 2000 | 90/10 | 0.41 | 3.8 | - |
| D2 | 14 | 10 | - | 0.16 | 4.0 | - |
| D5 | 9 | 2 | - | 0.27 | 4.0 | 8.16 |
| D8 | 35 | 2 | 25/67 | 0.93 | 3.1 | 6.0 |
| D7 | 12 | 28 | 76/21 | 0.31 | 3.1 | 6.11 |
| D4 | 5000 | 0 | 96/4 | 1.26 | 7.9 | 38.0 |
| D8 | 235 | 2630 | 1/99 | 1.73 | 3.0 | 4.8 |
| D15  D17 | 800  240 | 20  270 | 20/80  10/90 | 0.82  0.33 | 2.9  3.1 | 5.1 |
| D4 | 48 | 400 | - | 0.22 | 5.0 | - |
| D2 | 1 | 1 | - | 0.2 | 3.3 | 5.4 |
| D7 | 500 | 2 | 5/84 | 0.59 | 3.2 | 7.7 |
| D7 | 16 | 1 | - | 1.1 | 3.5 | - |
| D7 | 416 | 160 | 80/15 | 0.49 | 2.6 | 6.4 |
| D10 | 240 | 0 | 13/87 | 0.94 | 3.2 | - |
| D6 | 240 | 40 | 28/68 | 0.51 | 3.0 | - |
| D6 | 200 | 430 | 51/40 | 0.59 | 2.4 | 5.2 |
| D12  D13 | 18  12 | 1  - | 1/99  - | 0.75  0.50 | 2.9  3.3 | 4.9 |
| Non-neuroleptospirosis cases (*n=10*) | | | | | | |
| D4 | 0 | 0 | - | 0.15 | 3.8 | 3.6 |
| D1 | 0 | 1700 | - | 0.40 | 3.9 | 5.8 |
| D5 | 3 | 2000 | - | 0.27 | 4.1 | 7.1 |
| D5 | 3 | 1000 | - | - | - | 5.9 |
| D5 | 0 | 1000 | - | 0.24 | 4.2 | - |
| D3 | 1 | 400 | - | 0.22 | 3.9 | 9.5 |
| D4 | 4 | 0 | - | 0.23 | 3.3 | 6.2 |
| D5 | 6 | 12 | - | 0.22 | 4.5 | - |
| D2 | 2 | 1000 | - | - | - | - |
| D3 | 1 | 26 | - | 0.3 | - | - |

The table provides a case-by-case dataset of cerebrospinal fluid (CSF) parameters for all 28 patients who underwent a lumbar puncture during the study period, including both the 18 neuroleptospirosis cases and the 10 patients with non-neurological leptospirosis.

**Footnotes Definitions:**

^1^The day of lumbar puncture was from the first day of illness, defined as the day symptoms first appeared, regardless of the presence of fever or neurological signs.

The N/L ratio was not calculated when pleocytosis was insufficient or the sample was too hemorrhagic for differential cell counting.

Some data were unavailable because of clotted samples or technical issues in the laboratory.

**Abbreviations:** **CSF**, cerebrospinal fluid; **D**, Day; **IgG**, immunoglobulin G; **N/L**, Neutrophiles/Lymphocytes; **PMNs**, polymorphonuclear neutrophils; **RBC**, red blood cells; **WBC**, white blood cells.
