# Supplementary material for: Clinical features and outcomes of neuroleptospirosis in French Guiana: Insights from a retrospective study (2015–2021)
Source: PLoS Negl Trop Dis. 2026 Oct 15;20(10):e0014263. doi: 10.1371/journal.pntd.0014263 (PMC13690270; doi:10.1371/journal.pntd.0014263)
Supplement: Supplementary file 4 [file pntd.0014263.s004.docx]

**S4 Table. Comparison of cerebrospinal fluid parameters between patients with and without neuroleptospirosis.**

| CSF parameters | Neuroleptospirosis  (*n=20* lumbar punctures) | Non-neuroleptospirosis  (*n=*10 lumbar punctures) | p-value |
| --- | --- | --- | --- |
| CSF WBC/mm^3^ (*n=20*) | *n=20*, 42 [13–240] | *n=10*, 1.5 [0–3] | **0.001** |
| PMNs, % (*n=13*) | *n=13*, 25 [8–78] | - | - |
| Lymphocytes, % (*n=13*) | *n=13*, 68 [18–89] | - | - |
| CSF RBC/mm^3^ (*n=19*) | *n=19*, 10 [1–270] | *n=10*, 1000 [12–7000] | 0.167 |
| CSF protein, g/L (*n=20*) | *n=20*, 0.51 [0.32–0.88] | *n=8*, 0.235 [0.22–0.3] | **0.006** |
| CSF glucose, mmol/L (*n=20*) | *n=20*, 3.15 [3–3.66] | *n=7*, 3.91 [3.8–4.2] | **0.013** |
| CSF IgG, mg/L (*n=12*) | *n=12*, 86 [38–218] | *n=5*, 32 [14–34] | **0.020** |
| CSF lactate, mmol/L (*n=14*) | *n=14*, 2.5 [1.7–3.2] | *n=7*, 1.8 [1.6–1.9] | 0.071 |

The table presents a comparative statistical analysis of cerebrospinal (CSF) parameters, including leukocyte and red blood cell counts, protein, glucose, IgG, and lactate levels) between the neuroleptospirosis group (*n=*20 lumbar punctures from 18 patients) and the non-neuroleptospirosis group (*n=*10 lumbar punctures).

**Statistical Analysis:** P-values were calculated using the Mann-Whitney *U* test. Bold p-values indicate statistical significance (p ≤ 0.05).

**Abbreviations:** **CSF**, cerebrospinal fluid; **IgG**, immunoglobulin G; **IQR**, interquartile range; **RBC**, red blood cells; **WBC**, white blood cells.
