# Supplementary material for: Clinical features and outcomes of neuroleptospirosis in French Guiana: Insights from a retrospective study (2015–2021)
Source: PLoS Negl Trop Dis. 2026 Oct 15;20(10):e0014263. doi: 10.1371/journal.pntd.0014263 (PMC13690270; doi:10.1371/journal.pntd.0014263)
Supplement: Supplementary file 5 [file pntd.0014263.s005.docx]

**S5 Table. Cerebrospinal fluid leukocyte predominance according to the timing of lumbar puncture in patients with neuroleptospirosis.**

| D2 | Neutrophilic | D7 | Lymphocytic |
| --- | --- | --- | --- |
| D4 | Neutrophilic | D8 x 2 | Lymphocytic |
| D6 | Lymphocytic | D10 | Lymphocytic |
| D6 | Mixed | D12 | Lymphocytic |
| D7 | Neutrophilic | D15 | Lymphocytic |
| D7 | Neutrophilic | D17 | Lymphocytic |

The table shows the temporal evolution of CSF leukocyte predominance (neutrophilic, mixed, or lymphocytic) in the 18 patients with neuroleptospirosis, according to the number of days between illness onset and lumbar puncture.

**Onset of Illness:** The first day of illness was defined as the day symptoms first appeared, regardless of the presence of fever or neurological signs.

**Abbreviations:** **CSF**, cerebrospinal fluid; **D**, day.
