# Supplementary material for: Clinical features and outcomes of neuroleptospirosis in French Guiana: Insights from a retrospective study (2015–2021)
Source: PLoS Negl Trop Dis. 2026 Oct 15;20(10):e0014263. doi: 10.1371/journal.pntd.0014263 (PMC13690270; doi:10.1371/journal.pntd.0014263)
Supplement: Supplementary file 6 [file pntd.0014263.s006.docx]

**S6 Table. Distribution of MAT antibody titers and presumptive infecting serogroups.**

| **Neuroleptospirosis group: 23 positive MATs** | | | | | | | | | | |
| --- | --- | --- | --- | --- | --- | --- | --- | --- | --- | --- |
| Identified serogroup | Autumnalis | Ballum | Canicola | Copenhageni | Castellonis | Icterohaemorrhagiae | Patoc | Pyrogenes | Sejroe | Presumptive infecting serogroup |
| P1 | 100 |  | 400 |  |  |  | 400 | 800 |  | Pyrogenes |
| P2 |  |  | 3200 |  | 100 | 200 | 400 | 800 |  | Canicola |
| P3 |  |  | 1600 | 800 | 200 | 800 | 200 |  |  | Canicola |
| P4 |  |  |  |  |  | 3200 | 3200 |  |  | Icterohaemorrhagiae |
| P5 |  |  | 800 |  |  |  |  |  |  | Canicola |
| P6 |  |  | 3200 |  |  |  |  |  |  | Canicola |
| P7 |  |  |  | 3200 |  | 3200 |  |  |  | - |
| P8 |  |  |  |  |  | 800 |  |  |  | Icterohaemorrhagiae |
| P9 |  |  | 3200 |  |  | 3200 | 3200 |  |  | - |
| P10 |  |  |  |  |  | 800 |  |  |  | Icterohaemorrhagiae |
| P11 |  |  | 3200 |  |  |  |  |  |  | Canicola |
| P12 |  |  |  |  |  | 1600 |  |  |  | Icterohaemorrhagiae |
| P13 |  |  |  | 800 |  | 400 | 1600 |  |  | Copenhageni |
| P14 |  |  | 1600 |  |  |  |  |  |  | Canicola |
| P15 |  |  | 3200 |  |  |  |  |  |  | Canicola |
| P16 |  |  |  |  |  | 800 | 800 |  |  | Icterohaemorrhagiae |
| P17 |  |  | 3200 | 3200 |  | 3200 | 3200 |  |  | - |
| P18 |  |  |  | 3200 |  |  |  |  |  | Copenhageni |
| P19 |  |  | 400 | 1600 |  | 400 |  |  | 400 | Copenhageni |
| P20 |  | 800 |  |  |  |  | 200 |  |  | Ballum |
| P21 |  |  |  |  |  | 600 | 600 |  | 800 | Sejroe |
| P22 |  |  | 400 |  |  |  |  |  |  | Canicola |
| P23 |  |  | 3200 | 400 | 400 | 400 | 400 |  |  | Canicola |
| **Neuroleptospirosis group: 2 positive MATs** | | | | | | | | | | |
| P24 |  |  |  |  |  | 1600 |  |  |  | Icterohaemorrhagiae |
| P25 |  |  | 400 |  |  | 400 |  |  |  | - |

The table presents the individual microscopic agglutination test (MAT) antibody titers against the reference panel in the 25 patients in the study with a positive MAT result.

**Interpretation of Results:** Titers correspond to the highest serum dilution at which agglutination was observed. The presumptive infecting serogroup was defined by the highest MAT titer detected in the most recent sample. Co-agglutinins were defined as identical titers against multiple serogroups. **Abbreviations:** **MAT**, microscopic agglutination test; **P**, patient.
