# Supplementary material for: Clinical features and outcomes of neuroleptospirosis in French Guiana: Insights from a retrospective study (2015–2021)
Source: PLoS Negl Trop Dis. 2026 Oct 15;20(10):e0014263. doi: 10.1371/journal.pntd.0014263 (PMC13690270; doi:10.1371/journal.pntd.0014263)
Supplement: Supplementary file 7 [file pntd.0014263.s008.docx]

**RESPONSE TO REVIEWERS**

**Journal:** PLOS Neglected Tropical Diseases

**Manuscript ID:** PNTD-D-26-00737

**Title:** Clinical Features and Outcomes of Neuroleptospirosis in French Guiana: Insights from a Retrospective Study (2015–2021)

**Corresponding Author:** Dr. Audrey Delahaye

**Submission Deadline:** September 25, 2026

**Date of Revision:** September 5, 2026

Dear Dr. Brian Stevenson (Academic Editor), Dr. Joseph Vinetz (Section Editor), and members of the Editorial Board,

We sincerely thank you and the reviewers for the extremely thorough, encouraging, and constructive comments regarding our manuscript. We have carefully revised our paper to address every point raised. The comments have helped us to deeply strengthen the scientific and statistical rigor of our work, improve the precision of our diagnostics, and clarify crucial clinical nuances.

To address the concerns in full, we have notably:
1. Updated all biochemical and laboratory parameters to correct units of measurement (consistently using micromoles per liter, µmol/L, for creatinine and total bilirubin) and replaced outdated terms like acute renal failure with internationally standardized clinical terminology (AKI, Acute Kidney Injury).
2. Separated Table 3 into a highly comprehensive, readable two-part table, providing full case-by-case neurological, biological, and radiological data.
3. Fully addressed the selection and classification biases by expanding the Discussion on how our strict composite diagnostic criteria naturally excluded critical, thrombocytopenic, or unstable patients who had contraindications to lumbar punctures.
4. Downgraded our multivariable logistic regression model to a strictly exploratory, hypothesis-generating analysis to prevent overinterpretation given our sample size (18 cases), providing statistical explanations of the model's instability and wide 95% confidence intervals.
5. Reconciled our clinical and immunological timelines, explaining that neurological symptoms indeed occur early (direct neuroinvasion on Day 1), whereas positive serological markers on admission reflect the hospital presentation timeline (median delay of 5.5 days).
6. Addressed all Journal Requirements, providing an ethically secure Data Availability Statement, and detaching supplemental tables while listing their legends.

Below, we provide our detailed, point-by-point responses to the editors' and reviewers' comments, indicating exactly where the changes have been made in the marked-up copy of our revised manuscript ('Revised Manuscript with Track Changes').

# I. Response to Editorial and Journal Requirements

**Reviewer Comment:**
*1) Please ensure that the CRediT author contributions listed for every co-author are completed accurately and in full. At this stage, the following Authors require contributions: Audrey Delahaye, Tanguy Dequidt, Fabrice Quet, Clémentine Montagnac, Théo Blaise, Hatem Kallel, Félix Djossou, Mathieu Nacher, Kinda Shepers, Julien Coussement, Paul Le Turnier, and Loïc Epelboin.*

**Response:**
We have fully updated and completed the CRediT author contributions for all 12 co-authors in the 'Add/Edit/Remove Authors' section of the online submission form. For complete transparency, we have also listed the formal contributions in the Title Page of our revised manuscript body as follows:

• Audrey Delahaye (ADE): Conceptualization, Methodology, Project administration, Data curation, Formal analysis, Investigation, Writing – original draft.
• Tanguy Dequidt (TDE): Data curation, Formal analysis, Validation, Writing – review & editing.

• Fabrice Quet (FQU): Data curation, Formal analysis, Validation, Writing – review & editing.

• Clémentine Montagnac (CMO): Validation, Writing – review & editing.

• Théo Blaise (TBL): Project administration, Writing – review & editing.

• Hatem Kallel (HKA): Validation

• Félix Djossou (FDS): Validation

• Mathieu Nacher (MNA): Data curation, Formal analysis, Validation, Writing – review & editing.

• Kinda Schepers (KSC): Validation, Writing – review & editing.

• Julien Coussement (JCO): Validation, Writing – review & editing.
• Paul Le Turnier (PLT): Supervision, Validation, Writing – review & editing.

• Loïc Epelboin (LEP): Conceptualization, Methodology, Project administration, Supervision, Investigation, Data curation, Formal analysis, Validation, Writing – review & editing.

**Reviewer Comment:**
*5) We have noticed that you have uploaded Supporting Information files, but you have not included a list of legends. Please add a full list of legends for your Supporting Information files after the references list.*

**Response:**
In accordance with PLOS formatting guidelines, we have compiled and added a comprehensive 'Supporting Information Legends' section at the very end of our revised manuscript (following the References list, Page 19). The section lists detailed titles and descriptions for S1 Table through S6 Table.

**Reviewer Comment:**
*6) We notice that your supplementary Tables are included in the manuscript file. Please remove them and upload them with the file type 'Supporting Information'. Please ensure that each Supporting Information file has a legend listed in the manuscript after the references list.*

**Response:**
We have removed all supplemental tables from the main manuscript text. They have been formatted and saved as six separate, dedicated Word documents (labeled S1_Table.docx through S6_Table.docx) and uploaded individually under the 'Supporting Information' file type. We also corrected a minor header typo in S6_Table (which was mistakenly titled S5 Table internally).

**Reviewer Comment:**
*7) Please note that your Data Availability Statement is currently missing the DOI/accession number of each dataset OR a direct link to access each dataset.*

**Response:**
We acknowledge the journal's Open Science and data sharing policies. However, because our study involves sensitive clinical, demographic, and microbiological data from human subjects in a small, restricted geographical territory (French Guiana, with an approximate population of 300,000), public deposition of the raw individual-level dataset is ethically and legally restricted under the French National Data Protection Authority (CNIL) MR-004 ethical framework to protect patient privacy and prevent deductive re-identification.

To satisfy the journal's data access policies while ensuring absolute ethical compliance, we have updated our Data Availability Statement in the submission form. De-identified clinical data can be made available to qualified researchers upon reasonable request directed to a non-author institutional body. Our updated statement reads as follows:
"The individual clinical, biological, and radiological data underlying the findings of this study cannot be made publicly available due to ethical and legal restrictions regarding patient privacy and confidentiality, in compliance with the French Data Protection Authority (CNIL) MR-004 reference methodology. De-identified datasets can be made available to qualified researchers upon reasonable request. Data requests can be submitted to the Clinical Investigation Center of French Guiana (CIC Inserm 1424, Cayenne University Hospital, email: dric@ch-cayenne.fr) or to the local ethics committee."

# II. Response to Reviewer #1

**Reviewer Comment:**
*Lines 12–13: Please include more recent Brazilian studies on leptospirosis-associated meningitis to better contextualize the current knowledge and highlight the most up-to-date evidence from endemic settings.*

**Response:**
We thank the Reviewer for this valuable suggestion. In the revised manuscript, we have updated the second paragraph of the Introduction (Page 5) to include the most recent and relevant Brazilian studies on this topic. Specifically, we have cited the major meta-summary by Gadelha Farias et al. (PLOS Neglected Tropical Diseases, 2026) compiling 176 cases of leptospirosis-associated meningitis in urban endemic settings, alongside the study by Nabity et al. (Emerg Infect Dis, 2020) on anicteric meningitis. These references greatly strengthen the contextualization of our work within South American endemic settings.

**Reviewer Comment:**
*Lines 87–88: Please provide an appropriate reference to support this statement. A citation is needed to substantiate the information presented.*

**Response:**
We agree with the Reviewer. We have thoroughly supported this paragraph of the Introduction (Page 5) by adding robust, highly specific references to substantiate our statements regarding the risk of overlooked diagnoses:
1. The susceptibility of Leptospira in vitro to beta-lactams commonly used empirically is now supported by references to standard manuals.
2. The exclusion of Leptospira PCR from standard multiplex cerebrospinal fluid (CSF) panels is supported by references to international laboratory guidelines.
3. The clinical reality that patients might be cured empirically for suspected classic bacterial meningitis while their neuroleptospirosis goes overlooked is now supported by clinical series.

**Reviewer Comment:**
*Methodology - Please better specify the period, was it january 2015 until december 2021?*

**Response:**
We have clarified this in the revised manuscript. In the Methods section (Page 6, Line 140) and in the Abstract, we have explicitly specified that the study period ran from January 2015 to December 2021.

**Reviewer Comment:**
*Results - Some cases presented in Table 3 lack detailed descriptions of the neurological manifestations. Was the diagnosis based solely on the presence of fever and CSF findings, or did these patients also present neurological symptoms that were not reported? Since many cases were classified as meningeal, it would be helpful to provide additional details regarding the neurological presentation, particularly the presence of headache, nuchal stiffness, and other signs of meningeal irritation, whenever such information is available.*

**Response:**
We have fully addressed this comment by providing a highly detailed, case-by-case clinical description for all 18 patients in the newly expanded and updated Table 3 (Part 1, Lines 331).

In accordance with the Reviewer's excellent suggestion:
1. We have replaced any simplified syndromic labels with the exact clinical signs of meningeal irritation documented in the patients' medical charts at presentation (specifically detailing fever, headache, neck stiffness, vomiting, photophobia, and photophonophobia).
2. We have thoroughly audited every case to ensure they strictly satisfy our standardized protocol's clinical criteria for meningeal syndrome (requiring at least two clinical signs of meningeal irritation). For instance, the clinical description of Case 10 was revised to explicitly document the presence of both headaches and vomiting alongside fever, resolving any potential classification ambiguity.

The clinical presentation column now clearly demonstrates that the diagnosis of neuroleptospirosis was based on robust, detailed, and documented neurological symptoms in every single patient, well beyond isolated fever and abnormal CSF findings.

**Reviewer Comment:**
*Discussion - The sixth paragraph discussing CSF findings in neuroleptospirosis would benefit from a more comprehensive discussion. I suggest incorporating the findings of a recent meta-summary that provides a detailed overview of cerebrospinal fluid (CSF) patterns in neuroleptospirosis (Gadelha Farias et al., PLOS NTDs, 2026).*

**Response:**
We thank the Reviewer for highlighting this important publication, which we had not noticed. We have integrated a comprehensive comparison of our CSF results with the findings of the Gadelha Farias et al. (2026) meta-summary in the sixth paragraph of our revised Discussion (Page 18, Lines 350-351). Specifically, we have added and compared their reported mean values (such as a mean of 68 cells/mm³ with lymphocytic predominance) with our median values (42 cells/mm³ with 62% lymphocytic predominance). Discussing the wide dispersion of parameters in Gadelha Farias et al. allows us to emphasize to the reader that neither a marked pleocytosis nor an early neutrophilic predominance should rule out a diagnosis of neuroleptospirosis in endemic settings.

# III. Response to Reviewer #2

## Major Concerns

**Reviewer Comment:**
*1. The selection criteria for neuroleptospirosis cases might have influenced the outcome of this study. The definition may have preferentially selected patients with mild meningitic forms while excluding severe systemic cases in whom CSF examination could not be safely performed. In addition, the authors claim that neuroleptospirosis cases had a favorable clinical outcome. This is only true for neuroleptospirosis cases identified by your definition. The discussion needs to emphasize these issues.*

**Response:**
We thank the Reviewer for this major and highly constructive methodological comment. We completely agree that requiring cerebrospinal fluid (CSF) or radiological confirmation systematically introduces a classification and selection bias. In clinical practice, critically ill patients presenting with severe systemic complications (such as profound thrombocytopenia or hemodynamic instability) have formal contraindications to invasive lumbar punctures. Consequently, these severe cases were systematically excluded from our neuroleptospirosis group, even if neurological involvement was clinically suspected.

To ensure absolute scientific transparency and prevent any overinterpretation of our favorable clinical outcomes, we have significantly revised our manuscript:
1. In the Discussion (Page 18, Lines 349 and 357–358): We have added a comprehensive and detailed analysis of this selection bias. We explicitly discuss how severe thrombocytopenia and cardiovascular instability precluded lumbar punctures in the most critically ill patients, introducing a survivorship and selection bias that artificially shifted both our described clinical spectrum and our 100% survival rate toward a milder profile. We now clearly state that our findings strictly represent the prognosis of patients meeting our stringent, objective composite definition rather than the entire clinical spectrum of neurological leptospirosis.
2. In the Abstract (Page 3) and Author Summary (Page 4): We have also carefully qualified our prognosis statements. We have explicitly noted that the milder disease profile and excellent outcomes must be interpreted within the context of this hospital-managed cohort and our diagnostic inclusion criteria, which naturally excluded severe systemic presentations.

**Reviewer Comment:**
*2. The incidence estimated should be discussed as hospital-managed cases rather than population incidence and its implications.*

**Response:**
We agree with this point. In the revised Discussion (Page 17, Lines 346–347), we have explicitly specified that our annual incidence of 0.88 cases per 100,000 inhabitants strictly represents hospital-managed neuroleptospirosis and must not be interpreted as the true population-level incidence. We discuss that milder cases with transient or undocumented neurological symptoms managed entirely in outpatient settings are systematically missed by our hospital-based design. This means the actual public health burden is likely higher than reflected by hospital admission rates alone.

**Reviewer Comment:**
*3. Since there were only 18 neuroleptospirosis cases, modeling needs reevaluation and should discuss the limitations.*

**Response:**
We thank the Reviewer for this critical statistical point. In our revised manuscript, we have addressed the limitations of our model with absolute transparency:
1. We have added a dedicated paragraph in the statistical Limitations section of the Discussion (Page 19, Lines 358–359) explaining that our multivariable model (which retained three predictors for 18 events) violates the standard rule of 10 to 15 events per variable (EPV), leading to potential overfitting and statistical instability (evidenced by wide 95% CIs).
2. We have explicitly downgraded our multivariable findings in the Results (Page 16, Line 339) and Table 4 footnotes (Page 17, Line 344) to a strictly exploratory, hypothesis-generating analysis, emphasizing that larger prospective cohorts are mandatory to validate these predictors.
3. We have also restricted the use of continuous-to-categorical variables strictly to this exploratory logistic regression model, while ensuring that all bivariate comparisons (Table 1 and Table 2) utilize raw, continuous laboratory values to preserve statistical power.

**Reviewer Comment:**
*4. Age bias: is it a true bias?*

**Response:
Response:** We thank the Reviewer for this insightful query. We completely agree that framing the observed age difference primarily as a diagnostic or selection bias was too speculative.

In response to your comment and co-author consensus, we have removed these speculative bias hypotheses from the revised **Discussion (Page 18, Lines 355–356)**.

Instead, we have streamlined our Discussion to focus on the primary biological hypothesis: age-dependent variations in **blood-brain barrier (BBB) permeability** and host neuro-inflammatory response. Younger individuals may exhibit specific endothelial and BBB permeability dynamics that facilitate acute, early leptospiral neuroinvasion—a physiological mechanism well-recognized in other central nervous system infections and bacterial/aseptic meningitis, where age significantly influences host susceptibility and disease phenotype. We thank the Reviewer for encouraging us to simplify this section and remove unsupported speculative biases.

**Reviewer Comment:**
*5. Throughout the paper, researchers discuss how neuroleptospirosis patients have better clinical outcomes. However, in the discussion as well as the data analysis there is very little investigation into why this better outcome is seen. Is this truly due to differences in case presentation or could it be treatment, age, etc? Noted in table 2 there is a significant difference between the two groups in terms of the number of individuals that received IV antibiotics and meningeal dose antibiotic therapy. Another logistic regression model could be applied to determine what variables in the data best describe the differences in the outcomes of patients in neuroleptospirosis as well as non-leptospirosis patients.*

**Response:**
We sincerely thank the Reviewer for this interesting statistical suggestion. We have carefully evaluated the feasibility of performing a multivariable logistic regression model targeting clinical outcomes (such as ICU admission, death, or sepsis). However, we face major, insurmountable mathematical and statistical constraints that preclude the use of such modeling in our cohort:

1. The Issue of 'Perfect Separation' (Quasi-complete Separation): There were zero deaths (0/18, 0%) and zero ICU admissions (0/18, 0%) in the neuroleptospirosis cohort. Clinically, this highlights their uniformly excellent outcomes. Mathematically, however, when a predictor perfectly predicts the absence of an outcome (a zero-cell in the contingency table), maximum likelihood estimation for logistic regression fails to converge, yielding infinite odds ratios and uninterpretable standard errors.
2. Extreme Statistical Overfitting and Low Power: For the 'sepsis' outcome—which did occur in both groups—the neuroleptospirosis cohort only accounted for 9 events (9/18, 50%). Attempting to construct a multivariable model adjusting for critical clinical drivers such as age, intravenous antibiotic administration, and high 'meningitic' dosing would violate the fundamental statistical rule of 10 to 15 Events Per Variable (EPV). This would result in severe model overfitting and highly unstable estimates.

How we addressed this in the revised manuscript:
Rather than relying on unstable statistical models, we have thoroughly expanded and structured our Discussion (Page 18, Line 357) to analyze the clinical, demographic, and therapeutic drivers behind these favorable outcomes. We discuss that this excellent prognosis is multifactorial, driven by:
• Demographics: The significantly younger age of NL patients (median 26 vs. 38 years, p=0.001), youth being a well-established protective factor against severe leptospirosis.
• Phenotype (Primary Neuroleptospirosis): The clinical presentation was dominated by isolated, non-severe meningeal forms (78%), with a relative paucity of severe thrombocytopenia or hepatorenal dysfunction.
• Therapeutics: 100% of NL patients received early intravenous antibiotic therapy (vs. 79% in the non-NL group, p=0.042) and 59% received high, central nervous system-penetrating 'meningeal' doses, which likely aborted systemic bacterial replication and prevented clinical deterioration.

## Minor Concerns

**Reviewer Comment:**
*• Line 31: Extra spaces between misleading, clinical, and re-emerging.
• Line 129: Remove apostrophe after '(CDC)'.
• Line 342: ARC is used to define Acute renal failure, but later ARF is used in line 346.*

**Response:**
We thank the Reviewer for pointing out these typos. All of them have been corrected in the revised manuscript:
1. Spacing has been corrected in the abstract.
2. The apostrophe after (CDC) has been removed (Page 7).
3. Both ARC and ARF have been replaced throughout the manuscript and tables with the internationally accepted clinical standard abbreviation AKI (Acute Kidney Injury).

**Reviewer Comment:**
*• Line 46: 28 lumbar punctures are listed here; however, there are only 20 described in table 3 and only 18 patients are present in the neuroleptospirosis group.*

**Response:**
We have clarified this in the revised manuscript (Page 16, Line 336). A total of 28 lumbar punctures were performed across the entire cohort of 146 patients. Among them, 18 patients with objective CSF abnormalities met our strict case definition for neuroleptospirosis. The remaining 10 patients had clinical neurological symptoms but completely normal CSF parameters, and were therefore classified in the non-neuroleptospirosis group. To provide complete transparency, we have added two new supplemental tables (S3 Table and S4 Table) displaying individual CSF parameters for all 28 patients and a comparative statistical analysis of CSF features between both groups.

**Reviewer Comment:**
*• Line 51: p-value for age difference is listed at 0.001 in this line; however, the Table 1 and line 310 list the p-value as 0.002.*

**Response:**
We thank the Reviewer for spotting this discrepancy. The p-value for age compared as a continuous variable is 0.001, whereas the p-value for age compared as a categorical variable (categorized into three age groups) was 0.002. In the revised manuscript, we have removed the categorical p-value and report only the continuous age p-value (p=0.001) in Table 1 and throughout the text to ensure absolute mathematical consistency.

**Reviewer Comment:**
*• Line 216: What is defined at the 'first day of illness'? First day where fever is present?*

**Response:**
We have added a clear, explicit definition in the Methods section (Page 7) and in all relevant table footnotes: the onset of illness was defined as the first day any clinical symptom appeared, regardless of whether it was fever or a neurological symptom.

**Reviewer Comment:**
*• Line 142 and Line 247: You have 'less than fourfold increase' at line 142 but then in line 247 of Figure 1 it states 'fourfold increase'.*

**Response:**
We have corrected this discrepancy in Figure 1 to align perfectly with our diagnostic algorithm in S1 Table. Confirmed leptospirosis is strictly defined by a fourfold or greater increase in MAT titer, whereas probable leptospirosis is defined by a less than fourfold increase.

**Reviewer Comment:**
*• Table 3: Define 'Day of Lumbar puncture' is these days since hospitalization or days since first sign of illness?*

**Response:**
In Table 3 (Part 1, Footnote 1), we have explicitly defined that the day of lumbar puncture is calculated as the number of days elapsed since the first day of illness onset.

**Reviewer Comment:**
*• Table 3: A footnote 3 is listed on line 279 but no corresponding number is present in the table.*

**Response:**
We have corrected the footnote numbering in Table 3 (Part 1). Footnote 3 and all other footnotes are now sequentially and correctly ordered from 1 to 5.

**Reviewer Comment:**
*• Line 295: Was the patient with diffuse pachymeningitis tested for other common causes of pachymeningitis such as syphilis or tuberculosis?*

**Response:**
Yes, alternative infectious and inflammatory causes of pachymeningitis were thoroughly investigated and ruled out for this patient (Case 18). Specifically, neurosyphilis and Lyme neuroborreliosis were excluded via negative serological assays, tuberculosis was ruled out via negative CSF mycobacterial PCR and cultures, and sarcoidosis was evaluated and ruled out via normal serum angiotensin-converting enzyme levels. We have added this important clinical precision in the Results section (Page 16, Line 337) and in the legends of Figure 2.

**Reviewer Comment:**
*• Line 310-312: You indicate there is a significant numerical difference between the median WBC levels and creatinine levels. Is the difference between these numbers clinically relevant?*

**Response:**
We completely agree with the Reviewer. Although the difference in median values is statistically significant (WBC: 8.1 vs. 10.5 x 10⁹/L; creatinine: 92 vs. 110 µmol/L), the absolute clinical difference is indeed minimal and likely non-relevant on an individual patient level. We have added a discussion of this nuance in the Discussion section (Page 18, Line 349), explaining that these values should be interpreted collectively as a biological trend suggesting a less severe systemic inflammatory response and a milder systemic phenotype in patients with neuroleptospirosis.

**Reviewer Comment:**
*• Line 345: Generally, leukocytosis is defined as WBC count higher than 11 x 10^9/L.*

**Response:**
We have clarified in the footnotes of Table 4 (Page 17, Line 344) that we utilized a threshold of 10 x 10⁹/L to define leukocytosis in accordance with standard reference ranges used in local French medical laboratories.

**Reviewer Comment:**
*• Line 346: Is maximum creatinine >130 µmol/L a reliable indicator of acute renal failure?*

**Response:**
To prevent any diagnostic overstatement, we have replaced the term 'acute kidney injury' with 'creatinine level < 130 µmol/L' in Table 4 and its footnotes, ensuring that we only refer to objective biological cut-offs rather than syndromic definitions in our regression.

**Reviewer Comment:**
*• In Line 364- 366, 'Neurological manifestations were most often at disease onset in our series, thereby challenging the classical biphasic model that considers neuroleptospirosis as delayed immune complication,' but on lines 393-395 it is stated, ' This finding is consistent with the biphasic course of leptospirosis, in which neurological involvement preferentially occurs during the immune phase, when blood PCR sensitivity is reduced'*

**Response:**
We sincerely thank the Reviewer for identifying this logical inconsistency. We have fully resolved this point in our revised Discussion (Page 18, Line 352). Our clinical data show that neurological symptoms start early (during the acute bacteremic phase, on Day 1 of illness in 89% of cases), pointing toward direct early neuroinvasion. In contrast, the high rate of serological diagnoses at admission does not indicate a delayed clinical onset; rather, it is a biological consequence of the hospital presentation timeline. Since patients presented to the hospital with a median delay of 5.5 days, they were tested during the transition window when blood PCR sensitivity naturally declines while anti-Leptospira IgM titers rise. We have rewritten this paragraph to clarify how clinical onset (early) and biological testing (transition window) align, removing the contradictory statements.

**Reviewer Comment:**
*• Appendix 4: There are 25 patient serologies listed here, but there are only 18 patients in the neuroleptospirosis group. Are some of the serologies from the same patient?*

**Response:**
We have clarified this in the text (Page 16, Line 336). Only 25 Microscopic Agglutination Tests (MAT) were performed in the entire study cohort of 146 patients, among which only two were performed in the neuroleptospirosis group (S6 Table, formerly Appendix 4). This low number of MAT tests is a consequence of the withdrawal of national healthcare reimbursement for MAT in France in 2014, which we now explicitly discuss as a minor epidemiological limitation. The remaining 23 positive MAT serologies displayed in S6 Table correspond to patients from the non-neurological group.

# IV. Response to Reviewer #3

**Reviewer Comment:**
*• How were the patients informed about right to object to use of their data if retrospective? “All patients were informed about their right to object the use of their data.” Clarify.*

**Response:**
We have clarified our ethical procedures in the Ethics Statement of the Methods section (Page 6, Line 307). Since our retrospective study relied exclusively on routinely collected de-identified healthcare data, individual consent was not required under French regulations. However, to ensure absolute ethical compliance, all patients were informed about their right to object to the use of their data through a personalized letter accompanied by an objection form, supplemented by informational posters displayed across all participating clinical departments. No patient objected to the use of their data.

**Reviewer Comment:**
*• Line 137: Neuroleptospirosis definition is confusing. IgM seroconversion with a MAT titer ≥ 1:200, 2 IgM serologies ≥ 100 IU/mL performed at least seven days apart. Why 2 when one criterion is IgM > 50? What about a single MAT titer? Please reword and clarify.*

**Response:**
We thank the Reviewer for pointing out this confusion. Our case definitions strictly distinguish between 'confirmed' and 'probable' leptospirosis based on standard thresholds. To make our criteria immediately clear and readable, we have:
1. Rewritten and streamlined the text of our Case Definition in the Methods section (Page 7).
2. Added a new, comprehensive supplemental table (S1 Table) outlining the exact microbiological and serological thresholds.

Specifically, IgM levels ≥ 50 IU/mL (or seroconversion) are only considered 'confirmed' if accompanied by a high confirmatory MAT titer (≥ 1:200). If MAT is unavailable, a single high IgM ELISA ≥ 100 IU/mL is classified as a 'probable' case, whereas two high IgM ELISA tests ≥ 100 IU/mL taken at least seven days apart are required to reach 'confirmed' status.

**Reviewer Comment:**
*• Stats: Why were age and some variables categorized? For age, p-value was calculated for the continuous variable. Did you look at the raw uncategorized value for differences in comparisons?*

**Response:**
We thank the Reviewer for this statistical query. We have clarified our methodology in the Statistical Analysis section (Page 8, Line 155). To preserve statistical power and prevent any loss of clinical information, all bivariate comparisons of age and laboratory parameters reported in Table 1 and Table 2 were calculated using raw, continuous values (using the Mann-Whitney U test). Continuous-to-categorical transformation was strictly restricted to our exploratory multivariable logistic regression model (Table 4) to provide clinically interpretable odds ratios, which is standard practice in clinical research.

**Reviewer Comment:**
*• Conclusion: need rewording the following sentence. “Prospective collaborative studies base incorporating systematic CSF analysis and microbiological testing are warranted to further characterize the pathophysiology and optimize diagnostic strategies for neuroleptospirosis.”*

**Response:**
We have corrected and reworded this sentence in our revised Abstract (Page 3) and Discussion to improve clarity and remove the typographical error 'base':
"Future prospective cohorts incorporating systematic CSF and microbiological testing are warranted to clarify the pathophysiology and standardize diagnostic pathways."

**Reviewer Comment:**
*• Recruitment not clear: Study recruiting data from confirmed lepto during the study period? Should mention whether laboratory-confirmed lepto patients were recruited or not in the abstract.*

**Response:**
We have clarified this point in our revised Abstract (Page 3). The sentence now reads:
"We conducted a multicenter retrospective study across all public healthcare facilities of French Guiana (2015–2021) recruiting consecutive hospital-managed patients with laboratory-confirmed or probable leptospirosis."

**Reviewer Comment:**
*• Lines 362-363: difficult to comment about genetic susceptibility and sylvatic strains. Clarify.*

**Response:**
We completely agree with the Reviewer. Speculating on host genetic susceptibility or specific sylvatic strain neurotropism was unsupported by our data. In the revised manuscript, we have rewritten this paragraph in the Discussion (Page 17, Lines 348) to remove these hypotheses. We now state that the high proportion of gold miners and Brazilian-born patients in the neuroleptospirosis group is most pragmatically explained by their prolonged, high-risk occupational and environmental exposure to wild animal reservoirs in deep rainforest settings, which is well-documented in French Guiana.

**Reviewer Comment:**
*• When commenting about neuroleptospirosis presenting during early illness, it is important to consider that symptoms might not be apparent, and these are all retrospective. Can you correlate MAT positivity and PCR positivity in these patients on admission to see whether this statement can be supported from this study results?*

**Response:**
We thank the Reviewer for this highly rigorous physiological comment. We have thoroughly revised the Discussion (Page 18, Lines 353) to reconcile our clinical and biological timelines, supported by an admission-status correlation:
1. Early Clinical Presentation: Retrospective chart reviews confirm that neurological symptoms were highly apparent and objective from the very beginning, starting on Day 1 of illness in 89% (16/18) of our neuroleptospirosis cases. This strongly supports direct, early bacterial neuroinvasion during the acute leptospiremic phase.
2. Biological Correlation on Admission: To support this clinically, we analyzed the diagnostic markers on admission. Among our 18 cases, 61% (11/18) were confirmed (primarily via positive blood or CSF PCR during the acute bacteremic phase) and 39% (7/18) were probable (diagnosed via positive IgM serology), demonstrating that patients were caught precisely during the biological bacteremic-to-immune transition window.
3. The Presentation Delay Explanation: This biological distribution is perfectly explained by the hospital presentation timeline. The median delay between symptom onset and hospital encounter was 5.5 days (IQR 3–8). At this time point, blood PCR sensitivity naturally begins to decline while anti-Leptospira IgM titers rise.

**Reviewer Comment:**
*• CSF PCR not been performed is clearly a limitation.*

**Response:**
We completely agree. The lack of systematic CSF PCR testing is indeed a major diagnostic and microbiological limitation. We have explicitly added and discussed this limitation in two sections of our revised manuscript:
1. In the Results/Discussion (Page 18, Lines 354): We discuss that previous literature reports CSF PCR sensitivities of 60%–90% and that CSF bacterial loads can be up to 10-fold higher than in blood, sometimes remaining positive when blood PCR has cleared.
2. In the Limitations paragraph of the Discussion (Page 19, Lines 359): We have added a dedicated paragraph acknowledging that the lack of systematic CSF PCR may have led to missed diagnoses during the ultra-early phase of illness (before pleocytosis or IgM antibodies develop) and prevented absolute biological confirmation of direct CNS invasion in patients classified as 'probable' cases.

# V. Response to Reviewer #4

**Reviewer Comment:**
*1. The study objectives are not clearly stated. Suggest adding a couple of lines on this.*

**Response:**
We have revised the final paragraph of the Introduction (Page 6) to state our study objectives and underlying scientific hypothesis with absolute clarity:
1. Our primary objective is to characterize the epidemiology, clinical features, and outcomes of neuroleptospirosis in French Guiana.
2. Our secondary objective is to identify the sociodemographic, clinical, and laboratory factors associated with neurological involvement.
3. We have formulated our testable hypothesis: we hypothesized that applying a standardized composite definition in a comprehensive hospital-managed cohort would reveal a higher prevalence of milder, meningitis-dominated phenotypes than those described in highly selected ICU settings.

**Reviewer Comment:**
*2. Suggest keeping the neurological syndromes to meningitis, encephalitis or meningo-encephalitis. Others such as paresthesia, facial diplegia or hemiparesis are likely symptoms/ signs related to these.*

**Response:**
We completely agree with the Reviewer’s clinical assessment. Treating neurological signs (such as paresthesia or cranial nerve palsies) as standalone syndromes was biologically incorrect and created classification confusion.

In the revised manuscript, we have strictly restricted our syndromic classification to the three consensus syndromes (meningeal, encephalitic, and meningoencephalitic):
1. In the Abstract (Page 3) and Author Summary, we now report that clinical presentations were restricted to meningeal (78%), encephalitic (17%), and meningoencephalitic (5.5%) syndromes, achieving perfect mathematical consistency (14 + 3 + 1 = 18 cases).
2. In the Results section (Page 16), signs such as facial diplegia, hemiparesis, and paresthesias are now explicitly and systematically described as clinical signs or focal deficits occurring within or alongside these primary central/peripheral nervous system syndromes, rather than being counted as distinct syndromic entities.
3. In Table 3 (Part 1), the syndromic column has been updated to display only these three standardized categories (M, E, ME).

**Reviewer Comment:**
*3. Lumbar puncture was performed in 28/146 patients (and only 18 had neuroleptospirosis), but CSF data are reported only for the neuroleptospirosis cases (Table 2). Suggest report how many of the 28 LPs were in neuroleptospirosis vs non-neurological cases, and provide a table comparing CSF parameters between the groups for all 28 patients. Please describe why LP was done in those without neurological syndromes.*

**Response:**
We thank the Reviewer for this highly relevant suggestion. We have addressed this in the revised manuscript:
1. We have added a comprehensive comparison of CSF parameters in our supplemental S3 Table and S4 Table. S3 Table provides case-by-case raw data for all 28 patients (18 in the neuroleptospirosis group and 10 in the non-neurological group). S4 Table compares the median values and IQRs of CSF parameters (WBC count, RBC count, protein, glucose, IgG, and lactate) between both groups, showing a statistically significant elevation of WBC (p=0.001), protein (p=0.006), and IgG (p=0.020) in the neuroleptospirosis group.
2. In the Results (Page 16, Line 336) and Methods, we have clarified clinical decision-making. Lumbar punctures were performed exclusively in patients presenting with baseline clinical symptoms suggestive of neurological involvement (such as fever and acute headaches). The 10 patients classified in the non-neurological group did indeed have neurological symptoms, but because their CSF and radiological parameters were completely normal, they did not meet our composite criteria for a neuroleptospirosis case.

**Reviewer Comment:**
*4. Table 3 – it would have been better if neurological features of all the neuroleptospirosis cases were given.*

**Response:**
We have expanded and separated Table 3 into a highly detailed two-part table. In Table 3 (Part 1, Lines 331), we now provide an exhaustive, case-by-case clinical description of all neurological symptoms and signs documented at presentation for all 18 cases.

**Reviewer Comment:**
*5. The authors state that “Neurological manifestations were most often at disease onset in our series, thereby challenging the classical biphasic model that considers neuroleptospirosis as a delayed immune complication”. However, they contradict this later in discussion – lines 384-388; 392-395. This needs clarification/ reconciling.*

**Response:**
We thank the Reviewer for pointing out this logical inconsistency, which we have now fully resolved. Our clinical data indeed show that neurological symptoms started on Day 1 of illness in 89% of neuroleptospirosis cases, supporting early neuroinvasion rather than a delayed secondary immune complication. However, the high rate of serological diagnoses at admission does not imply a delayed clinical onset; rather, it is a consequence of the hospital presentation timeline. Since patients presented with a median delay of 5.5 days, they were tested during the transition window when blood PCR sensitivity is known to drop and IgM titers begin to rise.

We have revised the Discussion (Lines 457-465) to clarify this distinction and ensure absolute consistency throughout the manuscript. We have removed the sentence stating that our findings are 'consistent with the classical biphasic course' and replaced it with an explanation of how the clinical onset (early) and the biological testing window (transition phase) align.

**Reviewer Comment:**
*6. Patients with neuroleptospirosis had milder disease. This is not novel, and has been reported earlier from Sri Lanka and Laos. Suggest add these references - Perera et al. PLoS Negl Trop Dis 20(4): e0014183.; Dittrich et al. Lancet Glob Health. 2015;3(2):e104-12.*

**Response:**
We thank the Reviewer for this suggestion. In our revised Discussion (Page 18, Line 357), we have cited these two important papers to contextualize our prognosis findings. We discuss that our observation of a milder, meningitis-dominated phenotype carrying excellent outcomes is in perfect alignment with reports from Sri Lanka (Perera et al.) and Laos (Dittrich et al.), where leptospirosis cases presenting as aseptic meningitis also exhibited a less severe clinical course and fewer markers of systemic severity.

**Reviewer Comment:**
*7. Neuroleptospirosis patients received more antibiotics and higher “meningitic” doses; this could explain lower rates of sepsis and other better outcomes. Were any patients given steroids?*

**Response:**
We have added these details and integrated this discussion in our revised manuscript:
1. In the Results section (Page 16, Line 337), we have clarified that no patient in either the neuroleptospirosis or the non-neurological group received adjuvant corticosteroid therapy during their hospital stay.
2. In the Discussion (Page 18, Line 357), we have added a paragraph analyzing this therapeutic driver. While the role of adjuvant corticosteroids in neuroleptospirosis remains debated in the literature, our cohort demonstrates that in patients presenting with predominantly meningitic forms, a uniformly favorable outcome can be achieved with early and adequate high-dose intravenous antibiotic therapy alone, without the need for adjuvant steroids.

**Reviewer Comment:**
*8. Better outcomes may also reflect lack of other organ involvement (Primary Neuroleptospirosis). How many patients had other organ involvement? Data on non-neurological involvement should be added to Table 3.*

**Response:**
We completely agree with the Reviewer’s insightful comment. The excellent prognosis of our neuroleptospirosis cohort is indeed highly likely driven by a milder systemic phenotype and a relative lack of other severe organ involvements (the concept of 'Primary Neuroleptospirosis'), in contrast to the severe multi-organ failure classically seen in systemic leptospirosis.

Because Table 3 is already split into two highly detailed, multi-column parts focusing strictly on individual neurological, CSF, and neuroradiological profiles, adding further non-neurological columns would severely compromise its readability. Instead, we would like to highlight that Table 2 already provides a comprehensive, comparative, and statistically powered dataset of all systemic and non-neurological organ involvements (renal, hepatic, respiratory, and hematological) between the NL and non-NL groups.

To make this clinical reality immediately clear to the reader, we have integrated the following modifications into the text:
1. In the Results section (Page 16, Lines 340–341): We have added a sentence explicitly quantifying non-neurological organ involvements for our 18 NL patients, demonstrating the paucity of severe visceral complications (e.g., median creatinine was mild at 92 µmol/L, only 31% had jaundice, and only 6% had dyspnea).
2. In the Discussion section (Page 18, Lines 358–359): We have added a dedicated paragraph discussing the concept of 'primary neuroleptospirosis' (neurological symptoms dominating in the absence of severe multi-organ failure) to explain the uniformly favorable outcomes in our cohort compared to ICU series.

**Reviewer Comment:**
*9. CSF was required for diagnosis – this is likely to exclude severe cases and those with low platelets, biasing the study population towards milder disease.*

**Response:**
We completely agree with the Reviewer. The requirement of objective CSF and/or radiological abnormalities for our case definition constitutes a major selection bias. In clinical practice, severe systemic cases of leptospirosis are frequently accompanied by profound thrombocytopenia or hemodynamic instability, which represent absolute contraindications to performing an invasive lumbar puncture. These patients were therefore systematically excluded from our neuroleptospirosis group, even if neurological signs were clinically suspected. We have discussed this selection and classification bias in detail in our revised Discussion (Page 18, Line 357) and explicitly acknowledged that a survivorship bias cannot be excluded, as the most severe cases may have died or deteriorated before undergoing neurological investigations.
