# Supplementary material for: Clinical features and outcomes of neuroleptospirosis in French Guiana: Insights from a retrospective study (2015–2021)
Source: PLoS Negl Trop Dis. 2026 Oct 15;20(10):e0014263. doi: 10.1371/journal.pntd.0014263 (PMC13690270; doi:10.1371/journal.pntd.0014263)
Supplement: Peer review history

# Peer review: Decision Letter 0

**DOI:** 10.1371/journal.pntd.0014263.r001

**Authors:** Joseph Vinetz, Brian Stevenson

27 Jul 2026

Response to Reviewers Revised Manuscript with Track Changes Manuscript

Shaden Kamhawi

co-Editor-in-Chief

PLOS Neglected Tropical Diseases

orcid.org/0000-0003-4304-636XX

Paul Brindley

co-Editor-in-Chief

PLOS Neglected Tropical Diseases

orcid.org/0000-0003-1765-0002

**Additional Editor Comments (if provided):** **Journal Requirements:**

1) Please ensure that the CRediT author contributions listed for every co-author are completed accurately and in full.

At this stage, the following Authors/Authors require contributions: Audrey Delahaye, Tanguy Dequidt, Fabrice Quet, Clémentine Montagnac, Théo Blaise, Hatem Kallel, Félix Djossou, Mathieu Nacher, Kinda Shepers, Julien Coussement, Paul Le Turnier, and Loïc Epelboin. Please ensure that the full contributions of each author are acknowledged in the "Add/Edit/Remove Authors" section of our submission form.

The list of CRediT author contributions may be found here: https://journals.plos.org/plosntds/s/authorship#loc-author-contributions

2) We ask that a manuscript source file is provided at Revision. Please upload your manuscript file as a .doc, .docx, .rtf or .tex. If you are providing a .tex file, please upload it under the item type u2018LaTeX Source Fileu2019 and leave your .pdf version as the item type u2018Manuscriptu2019.

3) Please upload all main figures as separate Figure files in .tif or .eps format. For more information about how to convert and format your figure files please see our guidelines:

https://journals.plos.org/plosntds/s/figures

4) Please upload a copy of Figures FIG 1, and 2A-C which you refer to in your text on pages 16, and 22. Or, if the figure is no longer to be included as part of the submission please remove all reference to it within the text.

5) We have noticed that you have uploaded Supporting Information files, but you have not included a list of legends. Please add a full list of legends for your Supporting Information files after the references list.

6) We notice that your supplementary Tables are included in the manuscript file. Please remove them and upload them with the file type 'Supporting Information'. Please ensure that each Supporting Information file has a legend listed in the manuscript after the references list.

7)Please note that your Data Availability Statement is currently missing the DOI/accession number of each dataset OR a direct link to access each dataset. If your manuscript is accepted for publication, you will be asked to provide these details on a very short timeline. We therefore suggest that you provide this information now, though we will not hold up the peer review process if you are unable.

8)We have amended your Competing Interest statement to comply with journal style. We kindly ask that you double check the statement and let us know if anything is incorrect.

**Reviewers' comments:**

**Key Review Criteria Required for Acceptance?**

As you describe the new analyses required for acceptance, please consider the following:

**Methods**

-Are the objectives of the study clearly articulated with a clear testable hypothesis stated?

-Is the study design appropriate to address the stated objectives?

-Is the population clearly described and appropriate for the hypothesis being tested?

-Is the sample size sufficient to ensure adequate power to address the hypothesis being tested?

-Were correct statistical analysis used to support conclusions?

-Are there concerns about ethical or regulatory requirements being met?

Reviewer #1: -Are the objectives of the study clearly articulated with a clear testable hypothesis stated? yes

-Is the study design appropriate to address the stated objectives? yes

-Is the population clearly described and appropriate for the hypothesis being tested? yes

-Is the sample size sufficient to ensure adequate power to address the hypothesis being tested? yes

-Were correct statistical analysis used to support conclusions? yes

-Are there concerns about ethical or regulatory requirements being met? yes

Reviewer #2: -Are the objectives of the study clearly articulated with a clear testable hypothesis stated?-NA

-Is the study design appropriate to address the stated objectives?-OK

-Is the population clearly described and appropriate for the hypothesis being tested? OK

-Is the sample size sufficient to ensure adequate power to address the hypothesis being tested? NO

-Were correct statistical analysis used to support conclusions?- Needs to be addressed

-Are there concerns about ethical or regulatory requirements being met?-Yes

Reviewer #3: Comments mentioned here regarding methodology.

• How were the patients informed about right to object to use of their data if retrospective?

“All patients were informed about their right to object the use of their data.” Clarify.

• Line 137

Neuroleptospirosis definition is confusing.

IgM seroconversion with a MAT titer ≥ 1:200,

2 IgM serologies ≥ 100 IU/mL performed at least seven days apart. Why 2 when one criterion is IgM > 50

What about a single MAT titer?

Please reword and clarify.

• Stats

Why were age and some variables categorized? Did you look at the raw uncategorized value for differences in comparisons? “laboratory parameters, were categorized using”

Reviewer #4: The article provides detailed methodology, with case definitions, diagnostic methods described in detail, and the flowchart providing details regarding diagnosis of confirmed and probable cases. Results are clearly presented. There are no ethical concerns.

Limitations include the retrospective nature, with its inherent drawbacks.

Study sample is too small for multivariable analysis.

The study objectives are not clearly stated.

**********

**Results**

-Does the analysis presented match the analysis plan?

-Are the results clearly and completely presented?

-Are the figures (Tables, Images) of sufficient quality for clarity?

Reviewer #1: -Does the analysis presented match the analysis plan? yes

-Are the results clearly and completely presented? yes

-Are the figures (Tables, Images) of sufficient quality for clarity? yes

Reviewer #2: Does the analysis presented match the analysis plan?- Yes

-Are the results clearly and completely presented?- Yes-Yes

-Are the figures (Tables, Images) of sufficient quality for clarity?-Yes

Reviewer #3: • What is the median age comparison across the 2 groups?

• One patient presented with diplegia/ischaemic stroke. Were these manifestations after the diagnosis of leptospirosis or prior?

This patient with stroke, thrombus in MRI: could it be just an ischaemic stroke in a predisposed individual as an incidental occurrence? Need to discuss this in the discussion.

• CSF pleocytosis in 18. Lymphocytes in 8 /13. Is it out of 18?

• Onset of symptom: which symptom was considered? For NL- is it fever or neurology?

• Why was meningeal dose antibiotics given for NNL group? (n=2)

• ALT and AST are the accepted abbreviation. Same for ARF, as AKI is the standard abbreviation.

Case3: only neurology is parasthesia: was there an objective sensory loss? Only 14 cells in CSF (profile not given). CSF protein is normal. How definitive is the diagnosis?

Figure 1:

Need to reword 165 positive serologies (is it both IgM and MAT or what is exactly meant here?)

There are 2 text boxes with same criteria (bottom left and right in green) one should be NNL

Need a better figure legend.

Table 3; what is G/I?

• Day of lumbar puncture – is it from the day of illness or admission?

• Need CSF: serum glucose ratio.

• CSF white cells is the better variable label rather than CSF pleocytosis (this means high count).

• Show N/L % rather than CSF profile conclusion in the Table.

• Consider the neurological diagnosis to be the variable label rather than M/E. Some have had peripheral symptoms only.

• Could the lepto confirmation be added here?

• Need imaging findings.

Figure 2: specify the case number in the figure legend.

Table 4: typo in the foot note: is it ARC or ARF? Better use accepted abbreviations such as AKI.

Reviewer #4: The results are in keeping with the study plan. Results are clearly presented. Figures and table are clear.

**********

**Conclusions**

-Are the conclusions supported by the data presented?

-Are the limitations of analysis clearly described?

-Do the authors discuss how these data can be helpful to advance our understanding of the topic under study?

-Is public health relevance addressed?

Reviewer #1: -Are the conclusions supported by the data presented? yes

-Are the limitations of analysis clearly described? yes

-Do the authors discuss how these data can be helpful to advance our understanding of the topic under study? yes

-Is public health relevance addressed? yes

Reviewer #2: Are the conclusions supported by the data presented?- Needs addressing

-Are the limitations of analysis clearly described?- Needs addressing

-Do the authors discuss how these data can be helpful to advance our understanding of the topic under study?-Yes

-Is public health relevance addressed?-yes

Reviewer #3: Yes.

Reviewer #4: The authors acknowledge most of the limitations of the study. Conclusions support the data.

The manuscript addresses a topic of significant public health importance, especially in tropical countries, and adds to the limited literature in the field.

**********

**Editorial and Data Presentation Modifications?**

Use this section for editorial suggestions as well as relatively minor modifications of existing data that would enhance clarity. If the only modifications needed are minor and/or editorial, you may wish to recommend “Minor Revision” or “Accept”.

Reviewer #1: Dear authors,

The article is well written and provides relevant data from a multicenter retrospective study. I have only a few suggestions that I believe will further strengthen the manuscript and improve its clarity. Overall, I recommend acceptance after minor revisions addressing the comments above.

Abstract

The abstract is clear, well structured, and adequately summarizes the objectives, methods, main findings, and conclusions of the study. No major changes are required.

Introduction

Lines 12–13: Please include more recent Brazilian studies on leptospirosis-associated meningitis to better contextualize the current knowledge and highlight the most up-to-date evidence from endemic settings.

Lines 87–88: Please provide an appropriate reference to support this statement. A citation is needed to substantiate the information presented.

Methodology

- Please better specify the period, was it january 2015 until december 2021?

Results

- Some cases presented in Table 3 lack detailed descriptions of the neurological manifestations. Was the diagnosis based solely on the presence of fever and CSF findings, or did these patients also present neurological symptoms that were not reported? Since many cases were classified as meningeal, it would be helpful to provide additional details regarding the neurological presentation, particularly the presence of headache, nuchal stiffness, and other signs of meningeal irritation, whenever such information is available.

Discussion

- The sixth paragraph discussing CSF findings in neuroleptospirosis would benefit from a more comprehensive discussion. I suggest incorporating the findings of a recent meta-summary that provides a detailed overview of cerebrospinal fluid (CSF) patterns in neuroleptospirosis. The following study may be particularly relevant: Gadelha Farias LAB, Neto OMV, Sobrinho EPL, de Almeida Vale CL, Júnior GBS, de Francesco Daher E, de Alcântara GFT, Neto ASL, Coelho TMS, de Oliveira MS, Neto LVP. Leptospirosis-associated meningitis in an urban tropical endemic setting in northeastern Brazil: Three new cases and a meta-summary of 176 reported cases. PLoS Negl Trop Dis. 2026;20(5):e0014315. doi:10.1371/journal.pntd.0014315. This study summarizes the CSF characteristics reported across 176 published cases and could enrich the discussion by placing the authors' findings into the context of the existing literature.

Best regards

Reviewer #2: see below

Reviewer #3: General: Improve grammar

LINE 187: Duplicated “imaging, imaging”

Reviewer #4: 1. The study objectives are not clearly stated. Suggest adding a couple of lines on this.

2. Suggest keeping the neurological syndromes to meningitis, encephalitis or meningo-encephalitis. Others such as parasethesiae, facial diplegia or hemiparesis are likely symptoms/ signs related to these.

3. Lumbar puncture was performed in 28/146 patients (and only 18 had neuroleptospirosis) , but CSF data are reported only for the neuroleptospirosis cases (Table 2). Suggest report how many of the 28 LPs were in neuro-leptospirosis vs non-neurological cases, and provide a table comparing CSF parameters between the groups for all 28 patients. Please describe why LP was done in those without neurological syndromes.

4. Table 3 – it would have been better if neurological features of all the neuroleptospirosis cases were given.

5. The authors state that “Neurological manifestations were most often at disease onset in our series, thereby challenging the classical biphasic model that considers neuroleptospirosis as a delayed immune complication”. However, they contradict this later in discussion – lines 384-388; 392-395. This needs clarification/ reconciling.

6. Patients with neuroleptospirosis had milder disease. This is not novel, and has been reported earlier from Sri Lanka and Laos. Suggest add these references - Perera et al. PLoS Negl Trop Dis 20(4): e0014183.; Dittrich et al. Lancet Glob Health. 2015;3(2):e104-12.

7. Neuroleptospirosis patients received more antibiotics and higher “meningitic” doses; this could explain lower rates of sepsis and other better outcomes. Were any patients given steroids?

8. Better outcomes may also reflect lack of other organ involvement (Primary Neuroleptospirosis). How many patients had other organ involvement? Data on non-neurological involvement should be added to Table 3.

9. CSF was required for diagnosis – this is likely to exclude severe cases and those with low platelets, biasing the study population towards milder disease.

**********

**Summary and General Comments**

Use this section to provide overall comments, discuss strengths/weaknesses of the study, novelty, significance, general execution and scholarship. You may also include additional comments for the author, including concerns about dual publication, research ethics, or publication ethics. If requesting major revision, please articulate the new experiments that are needed.

Reviewer #1: Dear authors,

The article is well written and provides relevant data from a multicenter retrospective study. I have only a few suggestions that I believe will further strengthen the manuscript and improve its clarity. Overall, I recommend acceptance after minor revisions addressing the comments above.

Abstract

The abstract is clear, well structured, and adequately summarizes the objectives, methods, main findings, and conclusions of the study. No major changes are required.

Introduction

Lines 12–13: Please include more recent Brazilian studies on leptospirosis-associated meningitis to better contextualize the current knowledge and highlight the most up-to-date evidence from endemic settings.

Lines 87–88: Please provide an appropriate reference to support this statement. A citation is needed to substantiate the information presented.

Methodology

- Please better specify the period, was it january 2015 until december 2021?

Results

- Some cases presented in Table 3 lack detailed descriptions of the neurological manifestations. Was the diagnosis based solely on the presence of fever and CSF findings, or did these patients also present neurological symptoms that were not reported? Since many cases were classified as meningeal, it would be helpful to provide additional details regarding the neurological presentation, particularly the presence of headache, nuchal stiffness, and other signs of meningeal irritation, whenever such information is available.

Discussion

- The sixth paragraph discussing CSF findings in neuroleptospirosis would benefit from a more comprehensive discussion. I suggest incorporating the findings of a recent meta-summary that provides a detailed overview of cerebrospinal fluid (CSF) patterns in neuroleptospirosis. The following study may be particularly relevant: Gadelha Farias LAB, Neto OMV, Sobrinho EPL, de Almeida Vale CL, Júnior GBS, de Francesco Daher E, de Alcântara GFT, Neto ASL, Coelho TMS, de Oliveira MS, Neto LVP. Leptospirosis-associated meningitis in an urban tropical endemic setting in northeastern Brazil: Three new cases and a meta-summary of 176 reported cases. PLoS Negl Trop Dis. 2026;20(5):e0014315. doi:10.1371/journal.pntd.0014315. This study summarizes the CSF characteristics reported across 176 published cases and could enrich the discussion by placing the authors' findings into the context of the existing literature.

Best regards

Reviewer #2: This paper presents a retrospective study of clinical neuroleptospirosis cases versus non-neuroleptospirosis cases in French Guiana. Utilizing the gathered data, researchers aimed to characterize the epidemiology, clinical features, and outcomes of neuroleptospirosis in an endemic setting. While this manuscript investigates an underrecognized manifestation of leptospirosis, several methodological and interpretive concerns should be addressed. In summary, potential selection bias from the neuroleptospirosis definition, small sample size, instability of multivariable analyses, overinterpretation of favorable prognosis, and insufficient discussion of diagnostic bias need to be addressed.

Major concerns:

• The selection criteria for neuroleptospirosis cases might have influenced the outcome of this study. The definition may have preferentially selected patients with mild meningitic forms while excluding severe systemic cases in whom CSF examination could not be safely performed. In addition, the authors claim that neuroleptospirosis cases had a favorable clinical outcome. This is only true for neuroleptospirosis cases identified by your definition. The discussion needs to emphasize these issues.

• The incidence estimated should be discussed as hospital-managed cases rather than population incidence and its implications.

• Since there were only 18 neuroleptospirosis cases, modeling needs reevaluation and should discuss the limitations.

• Age bias: is it a true bias?

• Throughout the paper, researchers discuss how neuroleptospirosis patients have better clinical outcomes. However, in the discussion as well as the data analysis there is very little investigation into why this better outcome is seen. Is this truly due to differences in case presentation or could it be treatment, age, etc? Noted in table 2 there is a significant difference between the two groups in terms of the number of individuals that received IV antibiotics and meningeal dose antibiotic therapy. Another logistic regression model could be applied to determine what variables in the data best describe the differences in the outcomes of patients in neuroleptospirosis as well as non-leptospirosis patients.

Minor concerns:

• Line 31: Extra spaces between misleading, clinical, and re-emerging

• Line 46: 28 lumbar punctures are listed here; however, there are only 20 described in table 3 and only 18 patients are present in the neuroleptospirosis group

• Line 51: p-value for age difference is listed at 0.001 in this line; however, the Table 1 and line 310 list the p-value as 0.002

• Line 129: Remove apostrophe after “(CDC)”

• Line 216: What is defined at the “first day of illness”? First day where fever is present?

• Line 142 and Line 247: You have “less than fourfold increase” at line 142 but then in line 247 of Figure 1 it states “fourfold increase”

• Table 3: Define “Day of Lumbar puncture” is these days since hospitalization or days since first sign of illness?

• Table 3: A footnote 3 is listed on line 279 but no corresponding number is present in the table.

• Line 295: Was the patient with diffuse pachymeningitis tested for other common causes of pachymeningitis such as syphilis or tuberculosis?

• Line 310-312: You indicate there is a significant numerical difference between the median WBC levels and creatinine levels. Is the difference between these numbers clinically relevant?

• Line 342: ARC is used to define Acute renal failure, but later ARF is used in line 346

• Line 345: Generally, leukocytosis is defined as WBC count higher than 11 x 10^9/L

• Line 346: Is maximum creatinine >130 µmol/L a reliable indicator of acute renal failure?

• In Line 364- 366, “Neurological manifestations were most often at disease onset in our series, thereby challenging the classical biphasic model that considers neuroleptospirosis as delayed immune complication,” but on lines 393-395 it is stated, “ This finding is consistent with the biphasic course of leptospirosis, in which neurological involvement preferentially occurs during the immune phase, when blood PCR sensitivity is reduced”

• Appendix 4: There are 25 patient serologies listed here, but there are only 18 patients in the neuroleptospirosis group. Are some of the serologies from the same patient?

Reviewer #3: The manuscript addresses an important5 topic with limited available data. it is worth publishing after major revisions as discussed above. Also, further comments on the abstract and discussion is worth considering.

Abstract

• Conclusion: need rewording the following sentence.

“Prospective collaborative studies base incorporating systematic CSF analysis

and microbiological testing are warranted to further characterize the pathophysiology and optimize diagnostic strategies for neuroleptospirosis.”

• Recruitment not clear:

Study recruiting data from confirmed lepto during the study period? Should mention whether laboratory-confirmed lepto patients were recruited or not in the abstract.

Discussion

• Lines 362-363: difficult to comment about genetic susceptibility and sylvetic strains. Clarify.

• When commenting about neuroleptospirosis presenting during early illness, it is important to consider that symptoms might not be apparent and these are all retrospective. Can you correlate MAT positivity and PCR positivity in these patients on admission to see whether this statement can be supported from this study results?

• CSF PCR not been performed is clearly a limitation.

Reviewer #4: This multicenter study of hospital-managed leptospirosis in French Guiana identified 18/146 (12%) cases with a diagnosis of neuroleptospirosis (11 confirmed, 7 probable). The authors report that neuroleptospirosis cases were younger, presented predominantly with a meningeal syndrome, showed moderate lymphocytic CSF pleocytosis, and had favourable outcomes with no ICU admissions, deaths or residual disability.

The manuscript addresses a topic of significant public health importance, especially in tropical countries, and adds to the limited literature in the field.

Strengths of the study include the multicentre data collection from all public healthcare facilities, of all consecutive leptospirosis cases over 7 years. The article provides detailed methodology, with case definitions, diagnostic methods described in detail, and the flowchart providing details regarding diagnosis of confirmed and probable cases. Results are clearly presented. There are no ethical concerns.

Limitations include the retrospective nature, with its inherent drawbacks. Study sample was too small for multivariable analysis.

The article is well written. Addressing the above concerns is likely to improve the quality of the article.

**********

PLOS authors have the option to publish the peer review history of their article (what does this mean?). If published, this will include your full peer review and any attached files.

If you choose “no”, your identity will remain anonymous but your review may still be made public.

**Do you want your identity to be public for this peer review?**  For information about this choice, including consent withdrawal, please see our Privacy Policy.

Reviewer #1: **Yes:**  Luís Arthur Brasil Gadelha Farias

Reviewer #2: No

Reviewer #3: No

Reviewer #4: No

**Figure resubmission:**

**Reproducibility:**

To enhance the reproducibility of your results, we recommend that authors of applicable studies deposit laboratory protocols in protocols.io, where a protocol can be assigned its own identifier (DOI) such that it can be cited independently in the future. Additionally, PLOS ONE offers an option to publish peer-reviewed clinical study protocols. Read more information on sharing protocols at https://plos.org/protocols?utm_medium=editorial-email&utm_source=authorletters&utm_campaign=protocols

# Author comment: Author response to Decision Letter 1

**DOI:** 10.1371/journal.pntd.0014263.r002

21 Sep 2026

Attachment Submitted filename: Response_to_Reviewers.docx

# Editor report: Decision Letter 1

**DOI:** 10.1371/journal.pntd.0014263.r003

**Authors:** Joseph Vinetz, Brian Stevenson

29 Sep 2026

Dear Doctor Delahaye,

We are pleased to inform you that your manuscript 'Clinical Features and Outcomes of Neuroleptospirosis in French Guiana: Insights from a Retrospective Study (2015–2021)' has been provisionally accepted for publication in PLOS Neglected Tropical Diseases.

Before your manuscript can be formally accepted you will need to complete some formatting changes, which you will receive in a follow up email. A member of our team will be in touch with a set of requests.

Please note that your manuscript will not be scheduled for publication until you have made the required changes, so a swift response is appreciated.

IMPORTANT: The editorial review process is now complete. PLOS will only permit corrections to spelling, formatting or significant scientific errors from this point onwards. Requests for major changes, or any which affect the scientific understanding of your work, will cause delays to the publication date of your manuscript.

Should you, your institution's press office or the journal office choose to press release your paper, you will automatically be opted out of early publication. We ask that you notify us now if you or your institution is planning to press release the article. All press must be co-ordinated with PLOS.

Thank you again for supporting Open Access publishing; we are looking forward to publishing your work in PLOS Neglected Tropical Diseases.

Best regards,

Brian Stevenson, Ph.D.

Academic Editor

PLOS Neglected Tropical Diseases

Joseph Vinetz

Section Editor

PLOS Neglected Tropical Diseases

Shaden Kamhawi

co-Editor-in-Chief

PLOS Neglected Tropical Diseases

orcid.org/0000-0003-4304-636XX

Paul Brindley

co-Editor-in-Chief

PLOS Neglected Tropical Diseases

orcid.org/0000-0003-1765-0002

***********************************************************

p.p1 {margin: 0.0px 0.0px 0.0px 0.0px; line-height: 16.0px; font: 14.0px Arial; color: #323333; -webkit-text-stroke: #323333}span.s1 {font-kerning: none

# Editor report: Acceptance letter

**DOI:** 10.1371/journal.pntd.0014263.r004

**Authors:** Joseph Vinetz, Brian Stevenson

Dear Doctor Delahaye,

We are delighted to inform you that your manuscript, "Clinical Features and Outcomes of Neuroleptospirosis in French Guiana: Insights from a Retrospective Study (2015–2021)," has been formally accepted for publication in PLOS Neglected Tropical Diseases.

We have now passed your article onto the PLOS Production Department who will complete the rest of the publication process. All authors will receive a confirmation email upon publication.

The corresponding author will soon be receiving a typeset proof for review, to ensure errors have not been introduced during production. Please review the PDF proof of your manuscript carefully, as this is the last chance to correct any scientific or type-setting errors. Please note that major changes, or those which affect the scientific understanding of the work, will likely cause delays to the publication date of your manuscript. Note: Proofs for Front Matter articles (Editorial, Viewpoint, Symposium, Review, etc...) are generated on a different schedule and may not be made available as quickly.

Soon after your final files are uploaded, the early version of your manuscript will be published online unless you opted out of this process. The date of the early version will be your article's publication date. The final article will be published to the same URL, and all versions of the paper will be accessible to readers.

For Research Articles, you will receive an invoice from PLOS for your publication fee after your manuscript has reached the completed accept phase. If you receive an email requesting payment before acceptance or for any other service, this may be a phishing scheme. Learn how to identify phishing emails and protect your accounts at https://explore.plos.org/phishing.

Thank you again for supporting open-access publishing; we are looking forward to publishing your work in PLOS Neglected Tropical Diseases.

Best regards,

Shaden Kamhawi

co-Editor-in-Chief

PLOS Neglected Tropical Diseases

Paul Brindley

co-Editor-in-Chief

PLOS Neglected Tropical Diseases
